# Association of Integrating Mental Health Into Pediatric Primary Care at Federally Qualified Health Centers With Utilization and Follow-up Care

**DOI:** 10.1001/jamanetworkopen.2023.9990

**Published:** 2023-04-26

**Authors:** Jihye Kim, R. Christopher Sheldrick, Kerrin Gallagher, Megan H. Bair-Merritt, Michelle P. Durham, Emily Feinberg, Anita Morris, Megan B. Cole

**Affiliations:** 1 Department of Epidemiology and Biostatistics, University of Nevada Las Vegas School of Public Health, Las Vegas; 2 Department of Health Law, Policy, and Management, Boston University School of Public Health, Boston, Massachusetts; 3 Department of Pediatrics, Boston University Chobanian and Avedisian School of Medicine, Boston, Massachusetts; 4 Department of Pediatrics, Boston Medical Center, Boston, Massachusetts; 5 Department of Psychiatry, Boston University Chobanian and Avedisian School of Medicine, Boston, Massachusetts; 6 Department of Psychiatry, Boston Medical Center, Boston, Massachusetts; 7 Hassenfeld Child Health Innovation Institute, Department of Health Services Policy and Practice, Brown University School of Public Health, Providence, Rhode Island

## Abstract

**Importance:**

More than 1 in 5 children in low-income families report a mental health (MH) problem, yet most face barriers accessing MH services. Integrating MH services into primary care at pediatric practices such as federally qualified health centers (FQHCs) may address these barriers.

**Objective:**

To examine the association of a comprehensive MH integration model with health care utilization, psychotropic medication use, and MH follow-up care among Medicaid-enrolled children at FQHCs.

**Design, Setting, and Participants:**

This retrospective cohort study used Massachusetts claims data from 2014 to 2017 to conduct difference-in-differences (DID) analyses before vs after implementation of a complete FQHC-based MH integration model. The sample included Medicaid-enrolled children aged 3 to 17 years who received primary care at 3 intervention FQHCs or 6 geographically proximal nonintervention FQHCs in Massachusetts. Data were analyzed in July 2022.

**Exposures:**

Receipt of care at an FQHC implementing the Transforming and Expanding Access to Mental Health Care in Urban Pediatrics (TEAM UP) model, which began fully integrating MH care into pediatrics in mid-2016.

**Main Outcomes and Measures:**

Utilization outcomes included primary care visits, MH service visits, emergency department (ED) visits, inpatient admissions, and psychotropic medication use. Follow-up visits within 7 days of a MH-related ED visit or hospitalization were also examined.

**Results:**

Among the 20 170 unique children in the study sample, at baseline (2014), their mean (SD) age was 9.0 (4.1) years, and 4876 (51.2%) were female. In contrast to nonintervention FQHCs, TEAM UP was positively associated with primary care visits with MH diagnoses (DID, 4.35 visits per 1000 patients per quarter; 95% CI, 0.02 to 8.67 visits per 1000 patients per quarter) and MH service use (DID, 54.86 visits per 1000 patients per quarter; 95% CI, 1.29 to 108.43 visits per 1000 patients per quarter) and was negatively associated with rates of psychotropic medication use (DID, −0.4%; 95% CI −0.7% to −0.01%) and polypharmacy (DID, −0.3%; 95% CI, −0.4% to −0.1%). TEAM UP was positively associated with ED visits without MH diagnoses (DID, 9.45 visits per 1000 patients per quarter; 95% CI, 1.06 to 17.84 visits per 1000 patients per quarter), but was not significantly associated with ED visits with MH diagnoses. No statistically significant changes were observed in inpatient admissions, follow-up visits after MH ED visits, or follow-up visits after MH hospitalizations.

**Conclusions and Relevance:**

The first 1.5 years of MH integration enhanced access to pediatric MH services while limiting the use of psychotropic medications. Additional implementation time is necessary to determine whether these changes will translate into reductions in avoidable utilization.

## Key Points

**Question:**

Is mental health (MH) integration at federally qualified health centers (FQHCs) associated with changes in health care utilization, psychotropic medication use, and follow-up care among Medicaid-enrolled children?

**Findings:**

This cohort study included 20 170 Medicaid-enrolled children aged 3 to 17 years served by FQHCs. After 1.5 implementation years, children receiving care at FQHCs with MH integration, compared with nonintervention FQHCs, had relative increases in primary care visits with MH diagnoses, MH service use, and emergency department visits; decreases in psychotropic medication use; and no changes in inpatient admissions or follow-up care.

**Meaning:**

These findings suggest that integrating MH care into FQHCs may improve access to MH services among Medicaid-enrolled children.

## Teaser

This cohort study examines the association of a comprehensive mental health integration model with health care utilization, psychotropic medication use, and mental health follow-up care among Medicaid-enrolled children at federally qualified health centers.

## Introduction

Living in poverty is associated with increased risk for mental health (MH) disorders,^1,2^ with 22% to 38% of children in low-income families reporting a MH problem^3,4,5^ compared with 13% to 22% of the general US pediatric population.^3,6,7^ Despite well-established evidence-based treatments for MH disorders,^8^ fewer than one-half of children with MH needs receive treatment.^9,10,11,12^ Even when MH treatment is provided, it is often delayed several years after symptom onset,^13^ and its duration is insufficient.^14,15^ Treatment barriers include cost of care, stigma, and systemic-level barriers, such as lack of training and support for primary care practitioners and shortages of bilingual and racially or ethnically diverse clinicians.^1^

Integration of MH care into primary care pediatrics offers a promising model for increasing access to MH care.^16,17,18,19,20,21,22,23,24^ Most children in the US have regular access to primary care,^25^ and managing MH concerns in these settings can decrease stigma and increase accessibility for caregivers and children.^26^ Earlier intervention may also prevent hospitalizations and emergency department (ED) visits.^27^

Starting in mid-2016, 3 Massachusetts-based federally qualified health centers (FQHCs) began implementing Transforming and Expanding Access to Mental Health in Urban Pediatrics (TEAM UP),^28^ which is a comprehensive integrated MH care model that serves children from birth through young adulthood.^29^ As further described in a recent study of TEAM UP,^30^ TEAM UP focuses on promotion, prevention, and early identification of emerging MH issues, swift access to MH services, and psychiatric consultation for complex cases. Services are delivered by a team of MH clinicians, community health workers, and primary care clinicians, who implement the model within a learning community that includes technical assistance, clinical training, and an evaluation group to prove and improve the model.

A recent study^30^ using electronic medical records at TEAM UP FQHCs documented high rates of screening, effective use of warm hand-offs (ie, in-person transfer of care between 2 clinicians)^31^ to connect children to care, reductions in polypharmacy, and increases in diagnosis and treatment for attention-deficit/hyperactivity disorder, although the data were limited to TEAM UP electronic medical records, and, thus, no comparison groups were available. An additional study^32^ using claims data, which included the same 6 comparison sites, indicated that after 1.5 years of implementation, TEAM UP was associated with increases in all-cause primary care visits, especially among children with baseline MH conditions, with no changes in avoidable utilization. However, that study^32^ was limited to data from a single Medicaid managed care plan. We expand on this prior work by examining the universe of Medicaid enrollees served by TEAM UP, with nearly 10 times the study population size as examined in prior work, and by assessing additional utilization and follow-up care measures. Our objectives were 2 fold: to investigate the association between implementation of TEAM UP and (1) health care utilization for children, including hospitalizations, ED visits, primary care visits, MH service use, and psychotropic medication use; and (2) 2 quality-of-care–sensitive MH follow-up care measures.

## Methods

### Data Source, Study Sample, and Attribution

The primary data source was the 2014 to 2017 Massachusetts All Payer Claims Data (APCD), which included medical claims, pharmacy claims, and member files for children with public and private insurance. Medical claims were inclusive of encounter data. Our secondary data source was the 2017 American Community Survey,^33^ containing zip code–level sociodemographic information that was merged with member zip codes.

The study sample included children aged 3 to 17 years who received their primary care services at 1 of 9 FQHCs in Massachusetts, including 3 that implemented TEAM UP and 6 geographically proximal FQHC comparison sites (see the eAppendix in Supplement 1 for details on comparison site selection). To assign children to FQHCs, we used an intent-to-treat approach by assigning children to an FQHC according to where they received their primary care before TEAM UP was implemented, which reduced potential selection bias. Each child was attributed to a practice according to where they received the plurality of their primary care visits in the last 18 months, using National Provider Identifiers listed in the Massachusetts Registration of Provider Organizations.^34^ In instances of a tie, the child was attributed to the practice with the most recent visit. On the basis of this initial attribution, children were further included if they received at least 1 qualifying primary care visit at a qualifying FQHC and were enrolled in at least 8 of 10 quarters in the baseline period and 4 of 6 quarters in the postperiod. Quarters in which otherwise eligible children were unenrolled were excluded. We further excluded children with private insurance because of differential censoring in APCD data following the 2016 *Gobeille v Liberty Mutual Insurance Company* Supreme Court ruling.^35^

Boston University’s institutional review board deemed the study not human participants research and waived informed consent because data were available through public request, in accordance with 45 CFR §46. We followed the Strengthening the Reporting of Observational Studies in Epidemiology (STROBE) reporting guidelines.^36^

### Exposure Definition

The intervention group included children attributed to 1 of 3 TEAM UP FQHCs. Our comparison group included children attributed to 1 of 6 similar, non–TEAM UP FQHCs. Because TEAM UP was implemented incrementally throughout 2016, we defined the baseline period from January 2014 through December 2015 and the postperiod from January 2017 through December 2017.

### Outcomes

The first set of utilization outcomes included counts of 4 mutually exclusive visit types based on the Health Partner’s Total Cost of Care classification approach^37^: inpatient admissions, ED visits, primary care visits, and other outpatient visits. Each utilization outcome was further subclassified by whether visits included a primary MH diagnosis based on *International Classification of Diseases, Ninth Revision* and *International Statistical Classification of Diseases and Related Health Problems, Tenth Revision* codes.

Additional utilization outcomes included visits for MH services based on procedure and revenue codes, which were subclassified as therapy, testing, consultation, and screening visit types (eTable 1 in Supplement 1). We further examined 3 separate outcomes for psychotropic medication use: any filled psychotropic prescription within the month, any stimulant prescription fill, and any polypharmacy fill, indicated by 2 or more classes of psychotropic medications (eTable 2 in Supplement 1). Finally, we examined 2 quality-of-care–sensitive follow-up measures: having follow-up visit within 7 days after a (1) MH-related ED visit and (2) MH-related hospitalization.^38^ These outcomes were calculated conditional on having an ED visit or hospitalization and, thus, had different denominators compared with other study outcomes.

### Statistical Analysis

Sample characteristics at baseline were summarized using descriptive statistics and standardized differences. We conducted person-quarter-level multivariable difference-in-differences (DID) regression analyses using generalized estimating equations that compared changes in outcomes for the intervention-FQHC patients vs comparison-FQHC patients before (2014 quarter 1 to 2015 quarter 4) vs during (2017 quarter 1 to 2017 quarter 4) implementation of TEAM UP. A negative binomial distribution and log link were used to examine count outcomes (reported as the number of visits per 1000 patients per quarter), and a binomial distribution and logit link were used for binary outcomes, measured as a percentage change. The main parameter of interest was the interaction between TEAM UP status and an indicator for the postperiod, or the DID, which we reported as marginal effects. The secondary parameter of interest was overall longitudinal change, or change in outcomes in the postperiod vs the preperiod, to measure overall secular trends. All models included year and FQHC fixed effects and a vector of patient-level covariates, including age, sex, select clinical indicators (ie, any MH disorder or asthma, the most common chronic condition affecting children^39^), and patient zip code–level characteristics (household median income, percentage of residents from historically minoritized racial and ethnic groups, percentage of residents speaking language other than English, and percentage of residents with incomes less than the federal poverty level), with errors clustered at the patient level to account for repeated measures. To investigate changes for children with preexisting MH diagnoses, who may have been more likely to benefit from TEAM UP, additional analyses were conducted using a subsample of children with 1 or more MH diagnoses at baseline.

We conducted robustness checks and subgroup analyses. First, to test for parallel trends in outcomes between groups before implementation, we examined the interaction between linear quarter and TEAM UP status in the preperiod. Second, we conducted subgroup analyses by age group. Statistical significance was set at 2-sided *P* < .05. SAS statistical software version 9.4 (SAS Institute) and Stata statistical software version 16 (StataCorp) were used for analyses. Data were analyzed in July 2022.

## Results

### Study Sample

Our sample included 151 995 person-quarters, representing 20 170 unique children; there were 58 996 person-quarters in the TEAM UP intervention group and 92 999 person-quarters in the comparison group. In the first quarter of the baseline period, the mean (SD) age was 9.0 (4.1) years, and 4876 patients (51.2%) were female (Table 1). Patients attributed to TEAM UP FQHCs were more likely to have asthma, trauma-related or stressor-related disorders, and other mood disorders at baseline, and to live in zip codes with lower median income and higher percentages of residents who were from minoritized racial and ethnic groups and with English as a primary language. For all other characteristics, standardized differences were less than 0.1. Similar patterns were observed for children with a baseline MH diagnosis (eTable 3 in Supplement 1).

**Table 1.** Baseline Patient Characteristics Between TEAM UP and Comparison Site FQHCs

Characteristic	Patients, No. (%)^a^			Standard difference
	Full sample (N = 151 995 person-quarters)	TEAM UP FQHCs (n = 58 996 person-quarters)	Comparison group FQHCs (n = 92 999 person-quarters)	
Age, mean (SD), y	9.0 (4.1)	8.8 (4.1)	9.1 (4.2)	0.053
Sex				
Female	4876 (51.2)	1837 (51.4)	3039 (51.1)	−0.006
Male	4648 (48.8)	1738 (48.6)	2910 (48.9)	
Select conditions				
Asthma	2746 (28.8)	1292 (36.1)	1454 (24.4)	−0.257
Any mental health disorder	4485 (47.1)	1791 (50.1)	2694 (45.3)	−0.096
Trauma-related and stressor-related disorders	2053 (21.6)	882 (24.7)	1171 (19.7)	−0.120
Attention-deficit/hyperactivity disorder	1389 (14.6)	586 (16.4)	803 (13.5)	−0.081
Depressive disorders	1273 (13.4)	534 (14.9)	739 (12.4)	−0.073
Disruptive, impulse-control, and conduct disorders	1038 (10.9)	456 (12.8)	582 (9.8)	−0.094
Anxiety disorders	855 (9.0)	349 (9.8)	506 (8.5)	−0.044
Other mood disorders	620 (6.5)	291 (8.1)	329 (5.5)	−0.104
Autism spectrum disorder	234 (2.5)	88 (2.5)	146 (2.5)	0.000
Schizophrenia spectrum and other psychotic disorders	213 (2.2)	88 (2.5)	125 (2.1)	−0.024
Bipolar and related disorders	146 (1.5)	68 (1.9)	78 (1.3)	−0.047
Other mental health conditions	247 (2.6)	101 (2.8)	146 (2.5)	−0.023
Zip code–level characteristics, mean (SD)				
Median income in zip code, $	53 373 (19 059)	51 238 (15 796)	54 657 (20 670)	0.186
Minoritized racial and ethnic groups, % of residents	49.3 (21.7)	52.2 (23.7)	47.6 (20.3)	−0.207
With primary language other than English, % of residents	48.3 (20.1)	40.6 (9.7%)	52.8 (23.1)	0.689
Income below federal poverty level, % of residents	18.1 (6.7)	18.3 (6.0)	17.9 (7.1)	−0.066
Medicaid enrollment	9524 (100)	3575 (100)	5949 (100)	NA
FQHC is primary site of primary care	9524 (100)	3575 (100)	5949 (100)	NA

Abbreviations: FQHC, Federally Qualified Health Center; TEAM UP, Transforming and Expanding Access to Mental Health Care in Urban Pediatrics.

^a^ Our final sample throughout the whole study period included 151 995 person-quarters, where 58 996 person-quarters were in the intervention group and 92 999 person-quarters were in the comparison group. For baseline patient characteristics, only 2014 quarter 1 data were used, with 3575 persons from TEAM UP FQHCs and 5949 persons from comparison group FQHCs, for a total of 9524 persons.

### Association of TEAM UP With Changes in Outcomes: Full Study Sample

Over the study period, across all FQHCs in the study sample, primary care visits and ED visits decreased, on average (Table 2). However, decreases were smaller for TEAM UP patients, where TEAM UP was associated with a relative increase in primary care visits with MH diagnoses (DID, 4.35 visits per 1000 patients per quarter; 95% CI, 0.02-8.67 visits per 1000 patients per quarter) and a relative increase in all-cause ED visits (DID, 11.18 visits per 1000 patients per quarter; 95% CI, 2.43-19.93 visits per 1000 patients per quarter), particularly explained by visits without MH diagnoses (DID, 9.45 visits per 1000 patients per quarter; 95% CI, 1.06-17.84 visits per 1000 patients per quarter), compared with patients at comparison FQHCs. We found no association between TEAM UP and inpatient admissions (all cause or MH related).

**Table 2.** Association Between TEAM UP and Health Care Utilization and Quality-of-Care–Sensitive Measures Among Medicaid Enrollees^a^

Utilization category	Utilization, No. of visits/1000 patients/quarter					
	TEAM UP FQHCs		Comparison FQHCs		Longitudinal change, difference (95% CI)	DID, marginal effect (95% CI)
	Preperiod (n = 37 804)	Postperiod (n = 21 192)	Preperiod (n = 60 381)	Postperiod (n = 32 618)		
Avoidable utilization						
Inpatient admissions	6.6	6.6	6.2	6.1	−0.1 (−1.0 to 0.9)	0.02 (−1.73 to 1.76)
With MH diagnosis	2.4	3.2	1.8	2.3	0.6 (0.1 to 1.2)	0.09 (−0.92 to 1.11)
Without MH diagnosis	4.2	3.4	4.4	3.8	−0.7 (−1.4 to 0.0)	−0.35 (−1.71 to 1.01)
ED visits	116.6	119.9	130.8	123.0	−3.6 (−8.1 to 1.0)	11.18 (2.43 to 19.93)
With MH diagnosis	8.9	8.9	8.0	6.7	−0.8 (−2.5 to 0.9)	1.74 (−0.52 to 4.01)
Without MH diagnosis	107.7	111.0	122.8	116.3	−2.7 (−6.9 to 1.4)	9.45 (1.06 to 17.84)
Other utilization						
Primary care total visits	594.5	502.8	580.5	514.6	−76.0 (−85.0 to −67.0)	−24.43 (−43.92 to −4.94)
With MH diagnosis	31.9	27.4	30.9	23.1	−6.5 (−8.6 to −4.4)	4.35 (0.02 to 8.67)
Without MH diagnosis	562.6	475.4	549.7	491.5	−69.5 (−78.2 to −60.8)	−28.62 (−47.29 to −9.94)
MH service use						
Any service	1944.6	1906.2	1596.2	1497.0	−72.2 (−157.4 to 13.0)	54.86 (1.29 to 108.43)
Therapy	1527.1	1532.9	1247.5	1150.2	−54.2 (−129.6 to 21.1)	110.70 (57.58 to 163.82)
Consultation	240.8	219.6	192.5	213.3	4.7 (−12.9 to 22.2)	−43.81 (−54.18 to −33.43)
MH screening	167.0	147.7	148.6	125.9	−21.2 (−25.0 to −17.4)	6.18 (−3.06 to 15.43)
MH testing	9.6	6.0	7.7	7.6	−1.4 (−3.2 to 0.3)	−3.18 (−4.65 to −1.71)
Psychotropic medications, % of patients						
Any psychotropic medication use (>0% of days)	5.6	5.9	4.0	4.6	0.6 (0.3 to 0.8)	−0.4 (−0.7 to −0.01)
Any stimulant medication use (>0% of days)	3.4	3.6	2.2	2.8	0.5 (0.3 to 0.6)	−0.4 (−0.7 to −0.2)
Polypharmacy (≥2 psychotropic medications)	1.6	1.7	0.9	1.3	0.3 (0.2 to 0.4)	−0.3 (−0.4 to −0.1)
Quality-of-care–sensitive follow-up measures, % of patients						
Follow-up visit after MH ED visit (MH or self-harm diagnosis)^b^^,^^c^	67.2	69.9	57.6	67.3	6.9 (−1.1 to 14.9)	−6.7 (−23.0 to 9.6)
Follow-up visit after MH hospitalization (MH diagnosis)^b^^,^^d^	71.2	63.4	66.7	65.5	−4.1 (−16.3 to 8.1)	−0.8 (−25.4 to 23.8)

Abbreviations: DID, difference-in-differences; ED, emergency department; FQHC, federally qualified health center; MH, mental health; TEAM UP, Transforming and Expanding Access to Mental Health Care in Urban Pediatrics.

^a^ Preperiod and postperiod estimates represent the mean number of visits per 1000 patients per quarter in the preperiod (2014 to 2016 quarter 2) vs the postperiod (2016 quarter 3 to2017). Longitudinal changes are differences in the mean between preperiod and postperiod for the whole sample represented with 95% CI associated with the *t* test. The DID estimates represent the interaction term between TEAM UP status and postperiod status.

^b^ Refers to follow-up visit within 7 days of discharge (either outpatient or primary care visit with MH diagnosis).

^c^ The number of observations used in the analysis for follow-up visits after MH ED visit is 653 because the follow-up visits occurred conditional on having MH ED visits.

^d^ The number of observations used in the analysis for follow-up visits after MH hospitalization is 243 because the follow-up visits occurred conditional on having MH hospitalization.

Separately, MH service use decreased, on average, within our aggregate study population over the study period (Table 2). However, TEAM UP was associated with a relative increase in any MH service use (DID, 54.86 visits per 1000 patients per quarter; 95% CI, 1.29 to 108.43 visits per 1000 patients per quarter), which reflected increases in therapy visits (DID, 110.7 visits per 1000 patients per quarter; 95% CI, 57.58 to 163.82 visits per 1000 patients per quarter) and decreases in consultation visits (DID, −43.81 visits per 1000 patients per quarter; 95% CI, −54.18 to −33.43 visits per 1000 patients per quarter) and testing visits (DID, −3.18 visits per 1000 patients per quarter; 95% CI −4.65 to −1.71 visits per 1000 patients per quarter).

On average, across our full study population, psychotropic medication use, stimulant medication use, and polypharmacy all increased over the study period (Table 2). However, increases in psychotropic medication use were smaller for TEAM UP patients, who were 0.4% (95% CI, −0.7% to −0.01%) less likely to have any psychotropic medication, 0.4% (95% CI, −0.7% to −0.2%) less likely to have any stimulant use, and 0.3% (95% CI, −0.4% to −0.1%) less likely to have polypharmacy, compared with patients at comparison FQHCs, following TEAM UP implementation. TEAM UP was not associated with statistically significant changes in follow-up visits after MH ED visits or hospitalizations.

### Association of TEAM UP With Changes in Outcomes: Children With Baseline MH Diagnoses

On average, when examining children with a baseline MH diagnosis, across our full study population, ED visits, primary care visits, and MH service use decreased over time (Table 3). TEAM UP was not associated with a statistically significant change in inpatient admissions, but was positively associated with ED visits with MH diagnoses (DID, 12.07 visits; 95% CI, 2.85-21.29 visits). TEAM UP was also positively associated with primary care visits with a MH diagnosis, representing an additional 29.5 primary care visits per 1000 patients per quarter (95% CI, 12.34-46.66 visits per 1000 patients per quarter).

**Table 3.** Association Between TEAM UP and Health Care Utilization and Quality-of-Care–Sensitive Measures Among Medicaid Enrollees With Any MH Diagnosis at Baseline^a^

Utilization category	Utilization, No. of visits/1000 patients/quarter					
	TEAM UP FQHCs		Comparison FQHCs		Longitudinal change, difference (95% CI)	DID, marginal effect (95% CI)
	Preperiod (n = 13 583)	Postperiod (n = 5706)	Preperiod (n = 19 091)	Postperiod (n = 7796)		
Avoidable utilization						
Inpatient admissions	12.1	12.4	12.2	9.5	−1.4 (−4.0 to 1.1)	3.04 (−2.34 to 8.42)
With MH diagnosis	6.7	9.5	5.8	6.0	1.3 (−0.6 to 3.2)	2.12 (−1.73 to 5.97)
Without MH diagnosis	5.4	3.0	6.3	3.5	−2.7 (−4.3 to −1.1)	−0.17 (−3.56 to 3.22)
ED visits	161.5	154.0	187.7	157.3	−20.9 (−31.7 to −10.1)	25.85 (5.21 to 46.48)
With MH diagnosis	24.7	24.0	25.5	16.5	−5.4 (−11.0 to 0.1)	12.07 (2.85 to 21.29)
Without MH diagnosis	136.9	130.0	162.2	140.7	−15.5 (−24.5 to −6.5)	15.58 (−2.86 to 34.02)
Other utilization						
Primary care total visits	707.6	564.0	708.4	608.1	−118.6 (−137.6 to −99.6)	−39.37 (−79.81 to 1.08)
With MH diagnosis	88.9	67.8	97.6	55.8	−33.1 (−39.8 to −26.4)	29.50 (12.34 to 46.66)
Without MH diagnosis	618.7	496.1	610.8	552.3	−85.5 (−103.3 to −67.7)	−60.65 (−96.28 to −25.03)
MH service use						
Any service	5115.6	4519.1	4726.9	4066.3	−630.8 (−901.6 to −360.1)	186.57 (−6.13 to 379.27)
Therapy	4240.6	3796.5	3941.9	3344.0	−530.8 (−771.1 to −290.6)	309.80 (137.12 to 482.49)
Consultation	670.3	560.5	608.8	577.5	−64.1 (−120.7 to −7.4)	−90.44 (−124.32 to −56.56)
MH screening	178.1	147.9	152.3	124.3	−28.7 (−36.1 to −21.4)	3.24 (−14.81 to 21.29)
MH testing	26.6	14.2	24.0	20.5	−7.2 (−13.0 to −1.4)	−9.24 (−14.07 to −4.41)
Psychotropic medications, % of patients						
Any psychotropic medication use (>0% of days)	14.2	16.9	12.1	14.7	2.7 (2.0 to 3.3)	−0.3 (−1.3 to 0.7)
Any stimulant medication use (>0% of days)	9.2	10.7	6.8	9.4	2.1 (1.6 to 2.7)	−1.2 (−1.9 to −4.5)
Polypharmacy (≥2 psychotropic medications)	4.2	5.3	2.8	4.4	1.4 (1.0 to 1.8)	−0.7 (−1.2 to −0.2)
Quality-of-care–sensitive follow-up measures, % of patients						
Follow-up visit after MH ED visit (MH or self-harm diagnosis)^b^^,^^c^	67.2	75.8	57.6	75.9	14.2 (4.7 to 23.8)	−6.5 (−26.6 to 13.6)
Follow-up visit after MH hospitalization (MH diagnosis)^b^^,^^d^	71.2	64.7	66.7	77.1	2.3 (−11.0 to 15.6)	−12.0 (−41.7 to 17.6)

Abbreviations: DID, difference-in-differences; ED, emergency department; FQHC, federally qualified health center; MH, mental health; TEAM UP, Transforming and Expanding Access to Mental Health Care in Urban Pediatrics.

^a^ Preperiod and postperiod estimates represent the mean number of visits per 1000 patients per quarter in the preperiod (2014 to 2016 quarter 2) vs the postperiod (2016 quarter 3 to 2017). Longitudinal changes are differences in the mean between preperiod and postperiod for the whole sample represented with 95% CI associated with the *t* test. The DID estimates represent the interaction term between TEAM UP status and postperiod status.

^b^ Refers to follow-up visit within 7 days of discharge (either outpatient or primary care visit with MH diagnosis).

^c^ The number of observations used in the analysis for follow-up visits after MH ED visit is 573 because the follow-up visits occurred conditional on having MH ED visits.

^d^ The number of observations used in the analysis for follow-up visits after MH hospitalization is 216 because the follow-up visits occurred conditional on having MH hospitalization.

Rates of service use for every MH service category decreased over the study period, whereas rates of psychotropic medication use increased (Table 3). However, TEAM UP was associated with an additional 309.8 therapy visits per 1000 patients per quarter (95% CI, 137.12 to 482.49 visits per 1000 patients per quarter) and no statistically significant change in overall MH service use. TEAM UP patients were also 1.2% less likely to use stimulants (95% CI, −1.9% to −4.5%) and 0.7% less likely to have polypharmacy (95% CI, −1.2% to −0.2%) than patients at comparison FQHCs following TEAM UP implementation. Follow-up after ED visits increased over the study period, but there was no association with TEAM UP. No statistically significant trends or associations were observed for follow-up after inpatient hospitalizations.

### Robustness Checks and Subanalyses

In testing the parallel trends assumption for DID analyses, data showed no differential trends in outcomes between TEAM UP vs comparison FQHCs before TEAM UP implementation (eFigures 1-16 in Supplement 1). Furthermore, subgroup analyses suggested heterogeneity in effects by age group (eTable 4 in Supplement 1). For instance, TEAM UP was associated with an increase in primary care visits with MH diagnoses among those aged 5 to 12 years, increases in therapy among those aged 5 to 17 years, and increases in MH screening among those aged 3 to 4 years.

Post hoc, we explored whether associations between TEAM UP and MH service use were attributable to changes in treatment intensity vs the number of children treated (eTables 5-8 in Supplement 1). With the exception of MH testing, we found no statistically significant association between TEAM UP and the number of unique children using any MH service, suggesting that TEAM UP was primarily associated with changes in MH service intensity but not the number of children receiving each service. Our sample excluded children who were newly served by the FQHCs during the TEAM UP implementation period.

## Discussion

This cohort study provides evidence that, during a time when primary care visits and MH service use were otherwise declining, implementation of the TEAM UP pediatric MH integration model was associated with relative increases in rates of MH-related primary care visits and relative increases in overall MH service use relative to comparison FQHCs. Moreover, although pediatric psychotropic medication use increased systemwide, TEAM UP was associated with relative decreases in psychotropic medication use relative to comparison FQHCs. TEAM UP was not associated with reductions in avoidable utilization or increases in follow-up visits after MH-related ED visits or hospitalizations in the short-term. To our knowledge, this study represents the largest study of pediatric MH integration to date that applies causal inference methods.

These results add to the evidence base supporting MH integration in pediatric primary care settings. For example, the observed positive association between TEAM UP and MH-related primary care visits is consistent with prior research,^32,40,41,42^ including an earlier study of TEAM UP^32^ and a separate study of a different pediatric MH integration model.^41^ More engagement in primary care is generally desirable given widespread underengagement in care, and use of primary care offers an opportunity for the integrated team to identify and mitigate MH issues. Likewise, the positive association between TEAM UP and MH service use is also consistent with prior research,^43,44,45,46,47,48^ including 1 study^46^ that documented increased psychotherapy use after 5 years of implementation. Unlike much prior research, our findings included larger samples of children, used a comparison group and a preintervention period, and examined a wide set of study outcomes.

We further add to this existing evidence by differentiating among MH services (eg, therapy, consultation, testing, and screening), finding that the positive association between TEAM UP and MH service use was mainly explained by therapy. This may be explained by improvements in identification of MH needs^49^ associated with TEAM UP, as well as inclusion of MH clinicians and community health workers on care teams at TEAM UP FQHCs. In contrast, TEAM UP was associated with decreases in MH consultation and MH testing. This may be due to substitution effects (ie, engaging in therapy rather than consultation).

Our findings that TEAM UP was associated with decreases in medication use and polypharmacy are an important addition to the pediatric literature. Although adult collaborative MH care models have generally increased initiation of and adherence to psychotropic medications,^50,51,52^ findings in pediatrics are mixed.^43,46,53,54^ We urge caution when interpreting the relative decreases in psychotropic medication use and polypharmacy associated with TEAM UP, because this finding may or may not indicate optimal quality of care. Although some experts express concern that expanding MH services may lead to unnecessary prescribing within primary care settings,^55,56^ receiving psychotropic medications when clinically indicated is often crucial for treatment of MH problems.^57,58^ We speculate that the team-based approach to care within TEAM UP may augment availability of nonmedication treatment options for MH needs, such as therapy by the integrated MH clinician. It is feasible that some medical practitioners or families who require MH care may choose medication in the absence of other treatment options, but opt for therapy when it is readily available.

Finally, previous studies^59,60^ found that pediatric MH integration was associated with reduced avoidable utilization such as ED visits, which we did not find in our study. Differences in findings may be due to competing efforts across Massachusetts to reduce hospitalizations and ED use across all sites of care,^61,62,63^ where the mean number of pediatric MH-related ED visits has been decreasing over time in Massachusetts,^64^ compared with increasing trends in pediatric MH ED visits nationally.^65,66,67^ It may also take more study time for increased access to MH care to translate into reductions in ED visits or hospitalizations.

### Limitations

We note several limitations of this study. First, claims data are collected for billing purposes and are subject to missing or incomplete data, which may result in some missed diagnoses or missed visit types. In particular, diagnosis codes may be underreported, and specific procedures codes may be missing if not otherwise reimbursed or in instances where reimbursement is made through a bundled visit rate or capitated payment. However, given the DID approach, any impact of potential missingness on the study results is likely minimal and nonsystematic, because the data are unlikely to be differentially missing in the postperiod for the intervention vs comparison groups. Second, although services and prescriptions could be attributed to specific patients, they could not be attributed to site; thus, it is difficult to assess whether services or prescribing occurred in a FQHC site or an outside site. Third, privately insured children were excluded from our sample because of differential censoring in the APCD. FQHCs in our study may also have different characteristics than FQHCs nationally. Thus, the results might not generalize to the privately insured or to all FQHCs. Fourth, FQHC participation in TEAM UP was nonrandom. However, we observed baseline parallel trends for outcomes between intervention and comparison groups, we purposely selected FQHCs for the comparison group that were similar to TEAM UP FQHCs, and our analyses were conducted at the patient level, where it is unlikely that patients selected an FQHC because of TEAM UP. Fifth, we used generalized estimating equation models to obtain population-level estimates while accounting for individual-level correlations over time. However, this approach may be sensitive to missing or unbalanced data, where we assume data to be missing completely at random.^68^ Sixth, our study is limited to 1.5 years of implementation time; thus, the findings reflect short-term changes only. Further analyses will be conducted as more recent data become available. Despite these limitations, this study offers important evidence supporting MH integration into pediatrics. Although prior studies have documented positive results regarding MH symptom severity^44,45,69,70,71,72,73,74^ and early childhood development,^75^ many studies have relied on small sample sizes and many lacked a comparison group.

## Conclusions

After 1.5 years of implementation time, we found that TEAM UP was associated with relative increases in MH-related primary care visits among Medicaid-enrolled children, especially among children with a MH diagnosis at baseline, relative to children at comparison FQHCs. TEAM UP was further associated with increases in MH service use, including therapy, without increasing psychotropic medication use or polypharmacy. These findings suggest that expanding the TEAM UP model has the potential to increase primary care engagement for MH needs among Medicaid-enrolled children. Additional implementation time is necessary to determine whether these changes will translate into reductions in avoidable utilization and improved health outcomes. Nonetheless, the findings offer evidence to policy makers and health system leadership seeking models of care that may improve care engagement for low-income children with MH needs.

## Notes

Supplement 1. **eAppendix.** Selection Procedure of Six Comparison FQHCs **eTable 1.** Definitions of Study Outcomes **eTable 2.** Classification of Psychotropic Medication Types **eTable 3.** Baseline Patient Characteristics Among Those With Baseline Mental Health Diagnosis Between TEAM UP and Comparison FQHCs **eTable 4.** Association Between TEAM UP and Health Care Utilization and Follow-up Care Measures Among Medicaid Enrollees by Age Group: Difference-in-Differences Results **eTable 5. **Distribution of the Mean Number of Visits per Patient per Quarter **eTable 6. **Distribution of the Mean Number of Visits With Mental Health Service Use per Patient per Quarter **eTable 7.** Association Between TEAM UP and Whether or Not Patients Used “Any MH Service” Reported as %: Difference-in-Differences Results **eTable 8.** Association Between TEAM UP and Whether or Not Patients Had “Any MH Service” as % Among Medicaid Enrollees With Any Mental Health Diagnosis at Baseline: Difference-in-Differences Results **eFigure 1.** Time Trends in Inpatient Admissions Among Medicaid-Enrolled Children **eFigure 2.** Time Trends in Inpatient Admissions Among Medicaid-Enrolled Children With a Baseline Mental Health Diagnosis **eFigure 3.** Time Trends in Emergency Department Visits Among Medicaid-Enrolled Children **eFigure 4.** Time Trends in Emergency Department Visits Among Medicaid-Enrolled Children With a Baseline Mental Health Diagnosis **eFigure 5. **Time Trends in Primary Care Visits Among Medicaid-Enrolled Children **eFigure 6. **Time Trends in Primary Care Visits Among Medicaid-Enrolled Children With a Baseline Mental Health Diagnosis **eFigure 7. **Time Trends in Any Mental Health Service Utilization Among Medicaid-Enrolled Children **eFigure 8.** Time Trends in Mental Health Therapy Utilization Among Medicaid-Enrolled Children **eFigure 9.** Time Trends in Mental Health Consultation Utilization Among Medicaid-Enrolled Children **eFigure 10.** Time Trends in Mental Health Testing Utilization Among Medicaid-Enrolled Children **eFigure 11.** Time Trends in Mental Health Screening Utilization Among Medicaid-Enrolled Children **eFigure 12.** Time Trends of Any Psychotropic Medication Use (>0% of Days in Quarter) Among Medicaid-Enrolled Children **eFigure 13.** Time Trends of Any Stimulant Use Among Medicaid-Enrolled Children **eFigure 14.** Time Trends of Polypharmacy (Having > 1 Class of Psychotropic Medication) Among Medicaid-Enrolled Children **eFigure 15.** Time Trends of 7-Days FU After ED Visit for MH/Intentional Self-Harm DX (Primary) **eFigure 16.** Time Trends of 7-Days FU After Hospitalization for MH DX (Primary)

Supplement 2.
Data Sharing Statement
