# Supplementary material for: Association of Integrating Mental Health Into Pediatric Primary Care at Federally Qualified Health Centers With Utilization and Follow-up Care
Source: JAMA Netw Open. 2023 Apr 26;6(4):e239990. doi: 10.1001/jamanetworkopen.2023.9990 (PMC10134009; doi:10.1001/jamanetworkopen.2023.9990)
Supplement: Supplement 1. — eAppendix. Selection Procedure of Six Comparison FQHCs eTable 1. Definitions of Study Outcomes eTable 2. Classification of Psychotropic Medication Types eTable 3. Baseline Patient Characteristics Among Those With Baseline Mental Health Diagnosis Between TEAM UP and Comparison FQHCs eTable 4. Association Between TEAM UP and Health Care Utilization and Follow-up Care Measures Among Medicaid Enrollees by Age Group: Difference-in-Differences Results eTable 5. Distribution of the Mean Number of Visits per Patient per Quarter eTable 6. Distribution of the Mean Number of Visits With Mental Health Service Use per Patient per Quarter eTable 7. Association Between TEAM UP and Whether or Not Patients Used “Any MH Service” Reported as %: Difference-in-Differences Results eTable 8. Association Between TEAM UP and Whether or Not Patients Had “Any MH Service” as % Among Medicaid Enrollees With Any Mental Health Diagnosis at Baseline: Difference-in-Differences Results eFigure 1. Time Trends in Inpatient Admissions Among Medicaid-Enrolled Children eFigure 2. Time Trends in Inpatient Admissions Among Medicaid-Enrolled Children With a Baseline Mental Health Diagnosis eFigure 3. Time Trends in Emergency Department Visits Among Medicaid-Enrolled Children eFigure 4. Time Trends in Emergency Department Visits Among Medicaid-Enrolled Children With a Baseline Mental Health Diagnosis eFigure 5. Time Trends in Primary Care Visits Among Medicaid-Enrolled Children eFigure 6. Time Trends in Primary Care Visits Among Medicaid-Enrolled Children With a Baseline Mental Health Diagnosis eFigure 7. Time Trends in Any Mental Health Service Utilization Among Medicaid-Enrolled Children eFigure 8. Time Trends in Mental Health Therapy Utilization Among Medicaid-Enrolled Children eFigure 9. Time Trends in Mental Health Consultation Utilization Among Medicaid-Enrolled Children eFigure 10. Time Trends in Mental Health Testing Utilization Among Medicaid-Enrolled Children eFigure 11. Time Trends in Mental Healt [file jamanetwopen-e239990-s001.pdf]

Supplemental Online Content

Kim J, Sheldrick RC, Gallagher K, et al. Association of integrating mental health into pediatric primary care at federally qualified health centers with utilization and follow-up care. *JAMA Netw Open*. 2023;6(4):e239990. doi:10.1001/jamanetworkopen.2023.9990

eAppendix. Selection Procedure of Six Comparison FQHCs

eTable 1. Definitions of Study Outcomes

eTable 2. Classification of Psychotropic Medication Types

eTable 3. Baseline Patient Characteristics Among Those With Baseline Mental Health Diagnosis Between TEAM UP and Comparison FQHCs

eTable 4. Association Between TEAM UP and Health Care Utilization and Follow-up Care Measures Among Medicaid Enrollees by Age Group: Difference-in-Differences Results

eTable 5. Distribution of the Mean Number of Visits per Patient per Quarter

eTable 6. Distribution of the Mean Number of Visits With Mental Health Service Use per Patient per Quarter

eTable 7. Association Between TEAM UP and Whether or Not Patients Used “Any MH Service” Reported as %: Difference-in-Differences Results

eTable 8. Association Between TEAM UP and Whether or Not Patients Had “Any MH Service” as % Among Medicaid Enrollees With Any Mental Health Diagnosis at Baseline: Difference-in-Differences Results

eFigure 1. Time Trends in Inpatient Admissions Among Medicaid-Enrolled Children

eFigure 2. Time Trends in Inpatient Admissions Among Medicaid-Enrolled Children With a Baseline Mental Health Diagnosis

eFigure 3. Time Trends in Emergency Department Visits Among Medicaid-Enrolled Children

eFigure 4. Time Trends in Emergency Department Visits Among Medicaid-Enrolled Children With a Baseline Mental Health Diagnosis

eFigure 5. Time Trends in Primary Care Visits Among Medicaid-Enrolled Children

eFigure 6. Time Trends in Primary Care Visits Among Medicaid-Enrolled Children With a Baseline Mental Health Diagnosis

eFigure 7. Time Trends in Any Mental Health Service Utilization Among Medicaid-Enrolled Children

eFigure 8. Time Trends in Mental Health Therapy Utilization Among Medicaid-Enrolled Children

eFigure 9. Time Trends in Mental Health Consultation Utilization Among Medicaid-Enrolled Children

eFigure 10. Time Trends in Mental Health Testing Utilization Among Medicaid-Enrolled Children

eFigure 11. Time Trends in Mental Health Screening Utilization Among Medicaid-Enrolled Children

© 2023 Kim J et al. *JAMA Network Open.*

eFigure 12. Time Trends of Any Psychotropic Medication Use (>0% of Days in Quarter) Among Medicaid-Enrolled Children

eFigure 13. Time Trends of Any Stimulant Use Among Medicaid-Enrolled Children

eFigure 14. Time Trends of Polypharmacy (Having > 1 Class of Psychotropic Medication) Among Medicaid-Enrolled Children

eFigure 15. Time Trends of 7-Days FU After ED Visit for MH/Intentional Self-Harm DX (Primary)

eFigure 16. Time Trends of 7-Days FU After Hospitalization for MH DX (Primary)

This supplemental material has been provided by the authors to give readers additional information about their work.

eAppendix. Selection Procedure of Six Comparison FQHCs

Comparison FQHCs were selected by a third party (data vendor), in conjunction with our research team, based on key FQHC characteristics: geographic location (i.e. proximity to a TEAM UP FQHC), size (i.e. number of unique patients served), and patient demographics based on race/ethnicity (percent patients who are white, non-Hispanic; Black, non-Hispanic, Hispanic), age, sex, primary language, income level (percent below poverty level), and insurance coverage types (e.g., percent Medicaid-enrolled). This selection process was done using a matching algorithm. The pool of available comparison site FQHCs was limited to those in Massachusetts and within 10 miles of a TEAM UP FQHC. Using this approach, the comparison group was pre-specified: this selection process for comparison site FQHCs was conducted in 2017—the beginning of this research study and years before we initiated these analyses. Pre-selecting comparison site FQHCs allowed us to develop detailed directories of all providers at each FQHC and in turn, this allowed us to attribute children to these FQHCs in the claims data. The same set of comparison site FQHC was also used in an earlier TEAM UP manuscript (Cole et al., 2019) – for more details on the characteristics of TEAM UP vs comparison site FQHCs, please see Cole et al, 2019, supplemental appendix.

© 2023 Kim J et al. *JAMA Network Open.*

eTable 1. Definitions of Study Outcomes

Variable name	Description	Identification Codes
Utilization outcomes		
ED visits	Visits where patients receive care from emergency department	Revenue code (REV): 0450,0451,0452, 0453, 0454, 0455, 0456, 0457, 0458, 0459, 0981 Place of service (POS): 23 CPT code: 99281, 99282, 99283, 99284, 99285
Inpatient admissions	Visits where patients are admitted to the hospital and have at least one overnight stay	Admission date (ADMDAT): populated Discharge date (DISDAT): populated Revenue code (REV): 100-219 Place of service (POS): 21, 31, 32, 33, 34, 51, 56, 61 Bill type (BILLTYPE): 12, 12, 21, 22, 32, 41 Discharge status (DISSTATUS): 30
Primary care visits	Visits where patients receive care from primary care provider or have primary care services	Claim type (CLMTYP): 1 PCP provider indicator (PCP_IND): yes PCP service indicator (PCP_FLAG): yes
Mental health service use		
Any MH service	Use of any mental health service	Any mental health service based on CPT and REV codes below for sub-categories of MH service use
MH service: Therapy	Use of behavioral health intake or evalulation, psychotherapy, psychotherapy crisis or crisis intervention, secondary behavioral health service, group therapy, other therapeutic BH service, family training and counseling, or outpatient behavioral health services or accomodations	BH intake /evalulation: 90791; 90791 HA; 90792; 90801; 90802<br>Psychotherapy: 90832-90838; 90846-90847; 90849; 90804-90808; 90816; 90818; 90821<br>Psychotherapy crisis/crisis intervention: 90839-90840; H2011<br>Secondary BH service: 96150-96155<br>Group therapy: 90853<br>Other therapeutic BH service: H2019, H2020<br>Family training and counseling: T1027<br>Outpatient behavioral health services or accomodations: REV codes 900-919; 1000-1005
MH service: Consultation	Use of family consultation or outside consultation	Family consultation: 90887<br>Outside consultation: 90882; H0046; H0032
MH service: MH screening	Use of behavioral health screening	96110
MH service: MH testing	Use of behavioral health testing or psychological testing or assessment	MH testing: 96111<br>Psychological testing or assessment: 96101, 96102, 96103, 96105, 96111, 96116, 96118, 96119, 96120, 96130-96139, 96146
Psychotropic medication use		
Any psychotropic medication use (>0% of days)	Use of any psychotropic medication	National drug codes (NDC) for psychotropic medication listed in below supplemental table
Any stimulant medication use (>0% of days)	Use of any stimulant medication	National drug codes (NDC) for stimulant medication listed in below supplemental table

Polypharmacy (2+ psychotropic medications)	Use of 2 or more types of psychotropic medications	National drug codes (NDC) for psychotropic medication listed in below supplemental table
Quality of care		
Follow-up visit after MH ED visit (MH or self-harm DX)	The percentage of ED visits for members ages 6 to 21 with a principal diagnosis of mental illness or intentional self-harm, who had a follow-up visit for mental illness within 7 days of the ED visit	Revenue code (REV): 0450,0451,0452, 0453, 0454, 0455, 0456, 0457, 0458, 0459, 0981 Place of service (POS): 23 CPT code: 99281, 99282, 99283, 99284, 99285
Follow-up visit after MH hospitalization (MH DX)	The percentage of discharges for members 6 to 21 years of age who were hospitalized for treatment of selected mental illness diagnosis and who received a follow-up visit with a mental health practitioner within 7 days of discharge	Admission date (ADMDAT): populated Discharge date (DISDAT): populated Revenue code (REV): 100-219 Place of service (POS): 21, 31, 32, 33, 34, 51, 56, 61 Bill type (BILLTYPE): 12, 12, 21, 22, 32, 41 Discharge status (DISSTATUS): 30
Other		
Mental health diagnosis	Mental health diagnosis	Diagnosis code (DX): Any ICD9 or ICD10 code for mental health disorder based on 640 HEDIS codes (366 ICD-9 and 274 ICD-10)

Note: To establish inpatient visits, all claims during the inpatient stay are collapsed into a single visit. To establish all other visits, all claims from the same provider (rendering or billing) on the same day are collapsed. MH service use flags will overlay the utilization variables. For each visit, a 0 or 1 will indicate if any MH service was provided or if any of the specific MH services were provided. As with the utilization variables, when collapsed at the person-quarter level, each row of data will sum up the total number of each service provided in that quarter.

© 2023 Kim J et al. *JAMA Network Open.*

eTable 2. Classification of Psychotropic Medication Types

Class of drugs	Diagnosis	Medication names
Stimulants	Hyperactivity, inattention, impulsivity	Amphetamine/dextroamphetamine combination (Adderall), Amphetamine/dextroamphetamine combination (Adderall XR), Dexmethylphenidate (Focalin), Dexmethylphenidate (Focalin XR), Dextroamphetamine (Dexedrine), Dextroamphetamine (Dexedrine Spansule), Lisdexamfetamine (Vyvanse), Methylphenidate (Metadate ER, Metadate CD, Methylin ER, Ritalin LA, Ritalin SR), Methylphenidate (Ritalin, Methylin), Methylphenidate (Concerta)
SNRI	Inattention, hyperactivity, impulsivity (2nd line ADHD)	Atomoxetine (Strattera)
Atypical Antipsyhotic	Mania, psychosis, aggression	Aripiprazole (Abilify), Risperidone (Risperdal), Quetiapine (Seroquel), Olanzapine (Zyprexa), Ziprasidone (Geodon)
Typical Antipsychotics	Psychosis, aggression	Chlorpromazine (Thorazine), Haloperidol (Haldol), Clozapine (Clozaril)
1st Line Antidepressant SSRIs	Depression, anxiety	Fluoxetine (Prozac), Escitalopram (Lexapro), Citalopram (Celexa), Sertraline (Zoloft)
Atypical Antidepressants-SNRIs	Depression, anxiety	Bupropion XR (Wellbutrin XL), Bupropion SR (Wellbutrin SR), Bupropion (Wellbutrin), Venlafaxine (Effexor XR), Duloxetine (Cymbalta)
Tricyclic Antidepressants	Anxiety	Clomipramine (Anafranil)
Anxiolytic	Anxiety	Buspirone (BuSpar), Hydroxyzine (Vistaril), Clonazepam (Klonopin), Lorazepam (Ativan)
Alpha Agonists		Clonidine (Catapres), Clonidine (NOS), Guanfacine (NOS), Guanfacine (Intuniv), Guanfacine (Tenex), Clonidine (Kapvay)
Mood Stabilizers		Divalproex (Depakote), Divalproex (Depakote ER), Lamotrigine (Lamictal), Lithium carbonate (Eskalith, Eskalith CR, Lithobid)
Mood stabilizers/Aggression		Carbamazepine (Tegretol), Oxcarbazepine (Trileptal)
Other		Gabapentin (Neurontin) (Anticonvulsant, also used for pain, sleep in adolescents), Topiramate (Topamax) (Anticonvulsant, also used for pain, sleep in adolescents)

Note: Polypharmacy was defined as having 2 or more classes of prescription within a quarter.

© 2023 Kim J et al. *JAMA Network Open.*

eTable 3. Baseline Patient Characteristics Among Those With Baseline Mental Health Diagnosis Between TEAM UP and Comparison FQHCs

	Full Sample (N= 46,176 person-quarters)	TEAM UP FQHCs (N= 19,289 person-quarters)	Comparison Group FQHCs (N= 26,887 person-quarters)	Std. Diff.
**Age, Mean (SD)**	9.0 (3.9)	9.0 (3.8)	9.0 (4.0)	-0.019
**Female, N (%)**	1,425 (43.8%)	583 (43.8%)	842 (43.8%)	0.000
**Select conditions, N (%)**				
Any mental health disorder	3,251 (100%)	1,330 (100%)	1,921 (100%)	N/A
Asthma	1,139 (35.0%)	557 (41.9%)	582 (30.3%)	-0.243
Trauma- and stressor-related disorders	1,530 (47.1%)	649 (48.8%)	881 (45.9%)	-0.059
Attention Deficit Hyperactivity Disorder	1,228 (37.8%)	534 (40.2%)	694 (36.1%)	-0.083
Depressive disorders	977 (30.1%)	424 (31.9%)	553 (28.8%)	-0.067
Disruptive, impulse-control, and conduct disorder	921 (28.3%)	424 (31.9%)	497 (25.9%)	-0.133
Anxiety disorders	652 (20.1%)	287 (21.6%)	365 (19.0%)	-0.064
Other mood disorder	544 (16.7%)	259 (19.5%)	285 (14.8%)	-0.123
Autism spectrum disorder or Asperger’s syndrome	226 (7.0%)	85 (6.4%)	141 (7.3%)	0.038
Schizophrenia spectrum and other psychotic disorders	185 (5.7%)	79 (5.9%)	106 (5.5%)	-0.018
Bipolar and related disorders	134 (4.1%)	62 (4.7%)	72 (3.7%)	-0.046
Other mental health conditions	211 (6.5%)	84 (6.3%)	127 (6.6%)	0.012
**Zip-code level characteristics, Mean (SD)**				
Median income in zip code ($)	$54,055 ($19,698)	$51,500 ($16,503)	$55,823 ($21,462)	0.226
% non-White	49.5% (22.5%)	52.7% (24.0%)	47.2% (21.2%)	-0.241
% speaking language other than English	46.2% (19.5%)	40.0% (9.6%)	50.5% (23.1%)	0.594
% below poverty line	18.1% (6.7%)	18.4% (6.1%)	17.9% (7.0%)	-0.075
**Medicaid, N (%)**	3,251 (100%)	1,330 (100%)	1,921 (100%)	N/A
**FQHC is primary site of primary care, N (%)**	3,251 (100%)	1,330 (100%)	1,921 (100%)	N/A

Abbreviations: FQHC, Federally Qualified Health Center
Note: Our final sample throughout the whole study period included 46,176 person-quarters, where 19,289 person-quarters were in the treatment group and 26,887 person-quarters were in the comparison group. For baseline patient characteristics, only 2014q1 data were used, with 1,330 persons from TEAM UP FQHCs and 1,921 persons from comparison group FQHCs, making total 3,251 persons.

© 2023 Kim J et al. *JAMA Network Open.*

eTable 4. Association Between TEAM UP and Health Care Utilization and Follow-up Care Measures Among Medicaid Enrollees by Age Group: Difference-in-Differences Results

	TEAM UP FQHCs		Comparison FQHCs		DID	
	Pre	Post	Pre	Post	Marginal effect	95% CI
**N (person-quarters)**						
**Utilization, per 1000 patients/quarter**						
**Avoidable utilization**						
Inpatient admissions						
Age 3-4 years	6.3	3.4	4.0	7.7	-4.55***	[-6.97, -2.13]
Age 5-12 years	4.8	5.2	5.2	4.9	0.47	[-1.71, 2.64]
Age 13-17 years	11.2	11.2	9.9	7.8	1.82	[-3.03, 6.68]
With MH DX						
Age 3-4 years	0.7	0.7	0.1	1.2	-1.45	[-4.40, 1.50]
Age 5-12 years	2.0	2.2	1.7	2.0	-0.26	[-1.38, 0.87]
Age 13-17 years	4.4	6.5	3.2	3.5	1.18	[-1.88, 4.23]
Without MH DX						
Age 3-4 years	5.7	2.7	3.9	6.6	-3.94***	[-6.15, -1.72]
Age 5-12 years	2.8	2.9	3.5	2.9	0.73	[-1.19, 2.65]
Age 13-17 years	6.8	4.7	6.8	4.4	0.04	[-3.41, 3.49]
ED visits						
Age 3-4 years	136.1	140.8	161.2	156.8	7.26	[-20.68, 35.20]
Age 5-12 years	100.3	101.4	118.0	109.5	8.01	[-2.77, 18.78]
Age 13-17 years	143.7	147.1	142.3	133.8	11.55	[-6.89, 30.00]
With MH DX						
Age 3-4 years	1.9	3.4	3.2	2.3	3.23	[-3.05, 9.52]
Age 5-12 years	7.2	5.8	7.7	6.0	-0.15	[-2.64, 2.33]
Age 13-17 years	17.2	17.9	11.6	9.8	3.19	[-2.49, 8.87]
Without MH DX						
Age 3-4 years	134.2	137.4	158.0	154.5	4.52	[-22.74, 31.78]
Age 5-12 years	93.1	95.6	110.3	103.5	8.51	[-1.94, 18.96]
Age 13-17 years	126.5	129.2	130.6	124.0	9.74	[-7.61, 27.09]
**Other utilization**						
PC total visits						
Age 3-4 years	687.9	586.0	681.6	598.0	-22.71	[-89.75, 44.34]
Age 5-12 years	569.8	499.9	569.6	502.9	-2.39	[-28.69, 23.91]
Age 13-17 years	596.3	467.3	547.1	500.5	-79.36***	[-114.64, -44.08]
With MH DX						
Age 3-4 years	22.5	17.8	27.9	18.8	2.99	[-8.51, 14.49]
Age 5-12 years	35.1	31.9	33.3	24.4	8.56*	[2.04, 15.07]
Age 13-17 years	30.3	22.9	27.1	22.7	-3.56	[-10.05, 2.94]

© 2023 Kim J et al. *JAMA Network Open.*

Without MH DX						
Age 3-4 years	665.4	568.2	653.7	579.2	-26.34	[-91.03, 38.36]
Age 5-12 years	534.7	468.0	536.3	478.6	-9.81	[-34.81, 15.20]
Age 13-17 years	566.1	444.4	520.0	477.8	-76.06***	[-110.08, -42.05]
**Mental health (MH) service use, per 1000 patients/quarter**						
Any service						
Age 3-4 years	1118.6	1306.8	794.3	963.2	-134.21*	[-239.83, -28.58]
Age 5-12 years	2223.4	2143.1	1898.6	1756.9	85.74*	[0.40, 171.09]
Age 13-17 years	1784.9	1720.5	1377.6	1239.1	109.01*	[2.15, 215.88]
Therapy						
Age 3-4 years	741.6	962.7	495.0	631.0	-8.56	[-99.58, 82.47]
Age 5-12 years	1763.2	1716.3	1488.1	1361.1	101.38*	[19.81, 182.94]
Age 13-17 years	1445.3	1441.7	1139.2	978.1	210.65***	[103.70, 317.61]
Consultation						
Age 3-4 years	175.0	157.6	122.8	181.9	-75.20***	[-101.42, -48.97]
Age 5-12 years	282.0	266.3	246.0	256.7	-31.10***	[-48.40, -13.81]
Age 13-17 years	182.4	154.9	112.3	145.3	-30.48***	[-46.65, -14.31]
MH screening						
Age 3-4 years	193.4	180.1	169.1	144.2	36.03**	[12.96, 59.11]
Age 5-12 years	168.0	153.5	154.9	129.6	12.24	[-0.19, 24.66]
Age 13-17 years	148.3	119.9	122.7	110.9	-12.77	[-26.68, 1.15]
MH testing						
Age 3-4 years	8.6	6.4	7.4	6.1	-1.43	[-7.26, 4.41]
Age 5-12 years	10.2	7.0	9.6	9.5	-2.64*	[-4.97, -0.31]
Age 13-17 years	9.0	4.0	3.4	4.8	-4.93***	[-6.84, -3.02]
**Psychotropic medications, % patients**						
Any psychotropic medication use (>0% of days)						
Age 3-4 years	1.8%	1.1%	1.3%	0.8%	-0.1%	[-0.9%, 0.7%]
Age 5-12 years	6.3%	6.6%	4.1%	5.0%	-0.3%	[-0.9%, 0.2%]
Age 13-17 years	6.2%	7.1%	5.2%	5.6%	-0.5%	[-1.3%, 0.3%]
Any stimulant medication use (>0% of days)						
Age 3-4 years	0.6%	0.7%	0.7%	0.4%	0.4%	[-0.4%, 1.2%]
Age 5-12 years	4.4%	4.4%	2.6%	3.5%	-0.5%**	[-0.9%, -0.1%]
Age 13-17 years	2.7%	3.2%	2.2%	2.5%	-0.5%	[-1.1%, 0.01%]
Polypharmacy (2+ psychotropic medications)						
Age 3-4 years	0.1%	0.1%	0.2%	0.1%	0.1%	[-1.3%, 1.5%]

© 2023 Kim J et al. *JAMA Network Open.*

Age 5-12 years	1.6%	1.8%	0.8%	1.2%	-0.2%	[-0.5%, 0.1%]
Age 13-17 years	2.2%	2.3%	1.4%	2.0%	-0.6%**	[-1.1%, -0.2%]
**Follow-up care measures, % patients** Follow-up visit[a] after MH ED visit (MH or self-harm DX)[b]						
Age 3-4 years	0.0%	0.0%	0.0%	0.0%	No sample	
Age 5-12 years	70.7%	73.7%	60.3%	71.4%	-18.9%	[-44.3%, 6.5%]
Age 13-17 years	64.5%	67.3%	54.5%	63.8%	3.3%	[-17.5%, 24%]
Follow-up visit[a] after MH hospitalization (MH DX)[c]						
Age 3-4 years	0.0%	0.0%	0.0%	0.0%	No sample	
Age 5-12 years	76.5%	66.7%	71.1%	71.4%	6.5%	[-22.6%, 35.6%]
Age 13-17 years	65.6%	60.9%	62.8%	59.3%	-15.2%	[-50.9%, 20.5%]

© 2023 Kim J et al. *JAMA Network Open.*

eTable 5. Distribution of the Mean Number of Visits per Patient per Quarter

# admissions/visits per quarter	Inpatient admissions		Emergency department visits		Primary care visits	
	Frequency	Percent	Frequency	Percent	Frequency	Percent
0	151,109	99.42	136,818	90.01	91,941	60.49
1	824	0.54	12,495	8.22	42,824	28.17
2	51	0.03	2,072	1.36	12,118	7.97
3	7	0	419	0.28	3,461	2.28
4	2	0	103	0.07	1,096	0.72
> 4	2	0	88	0.05	555	0.37
Total	151,995	100	151,995	100	151,995	100

Note: Among 151,995 person-quarters in our final data, the table shows the distribution of sample by the number of healthcare utilization per quarter for inpatient admission, emergency department visit, and primary care visit.

© 2023 Kim J et al. *JAMA Network Open.*

eTable 6. Distribution of the Mean Number of Visits With Mental Health Service Use per Patient per Quarter

# mental health service visits (any service) per quarter	Freq.	Percent
0	81,057	53.33
1	33,095	21.77
2	13,440	8.84
3	6,163	4.05
4	3,645	2.4
5	2,221	1.46
6	1,596	1.05
7	1,236	0.81
8	1,023	0.67
9	830	0.55
10	702	0.46
11	608	0.4
12	594	0.39
13	502	0.33
14	446	0.29
15	349	0.23
16	325	0.21
17	272	0.18
18	281	0.18
19	212	0.14
20	181	0.12
21	197	0.13
22	205	0.13
23	154	0.1
24	155	0.1
25	132	0.09
26	148	0.1
27	117	0.08
28	119	0.08
29	118	0.08
30	109	0.07
> 30	1763	1.07
Total	151,995	100

Note: Among 151,995 person-quarters in our final data, the table shows the distribution of sample by the number of any mental health service uses per quarter.

© 2023 Kim J et al. *JAMA Network Open.*

eTable 7. Association Between TEAM UP and Whether or Not Patients Used "Any MH Service" Reported as %: Difference-in-Differences Results

	TEAM UP FQHCs		Comparison FQHCs		DID	
	Pre	Post	Pre	Post	Marginal effect	95% CI
**N**	37,804	21,192	60,381	32,618		
**MH service use**						
Any service	27.0%	24.5%	23.0%	20.4%	0.3%	[-0.5%, 1.2%]
Therapy	12.4%	11.6%	9.3%	9.1%	-0.4%	[-0.9%, 0.2%]
Consultation	4.6%	4.8%	3.7%	4.0%	-0.2%	[-0.6%, 0.2%]
MH screening	16.5%	14.6%	14.8%	12.4%	0.7%	[-0.1%, 1.5%]
MH testing	0.4%	0.3%	0.4%	0.4%	-0.2%**	[-0.3%, -0.1%]

Note: Pre- and post-estimates represent the % of having each MH service in the pre (2014-q22016)- versus the post (q32016-2017)-periods. The difference-in-difference estimates represent the interaction term between TEAM UP status and post-period status. 95% confidence interval (CI) in brackets. MH is mental health.
** $p < 0.05$, ** $p < 0.01$, *** $p < 0.001$*

eTable 8. Association Between TEAM UP and Whether or Not Patients Had “Any MH Service” as % Among Medicaid Enrollees With Any Mental Health Diagnosis at Baseline: Difference-in-Differences Results

	TEAM UP FQHCs		Comparison FQHCs		DID	
	Pre	Post	Pre	Post	Marginal effect	95% CI
**N**	13,583	5,706	19,091	7,796		
**MH service use**						
Any service	46.5%	37.5%	41.0%	32.7%	-0.4%	[-2.3%, 1.4%]
Therapy	34.1%	26.9%	29.4%	22.9%	-0.2%	[-1.7%, 1.4%]
Consultation	12.9%	11.6%	11.7%	10.3%	-0.1%	[-1.2%, 1.1%]
MH screening	17.6%	14.7%	15.1%	12.3%	0.4%	[-1.1%, 1.9%]
MH testing	1.2%	0.6%	1.2%	1.0%	-0.4%*	[-0.7%, -0.1%]

Note: Pre- and post-estimates represent the % of having each MH service in the pre (2014-q22016)- versus the post (q32016-2017)-periods. The difference-in-difference estimates represent the interaction term between TEAM UP status and post-period status. 95% confidence interval (CI) in brackets. MH is mental health.
* $p < 0.05$, ** $p < 0.01$, *** $p < 0.001$

© 2023 Kim J et al. *JAMA Network Open.*

eFigure 1. Time Trends in Inpatient Admissions Among Medicaid-Enrolled Children

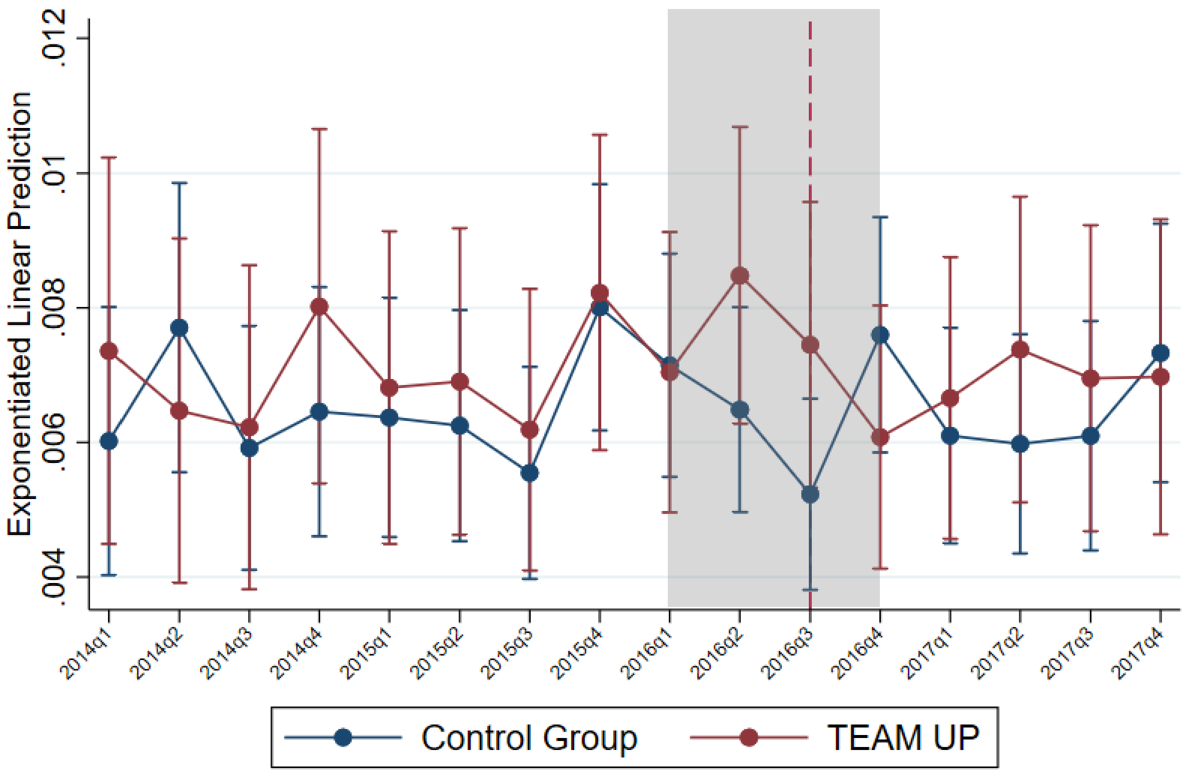

Note: This graph shows time trend in inpatient admissions among Medicaid-enrolled children.

© 2023 Kim J et al. *JAMA Network Open.*

eFigure 2. Time Trends in Inpatient Admissions Among Medicaid-Enrolled Children With a Baseline Mental Health Diagnosis

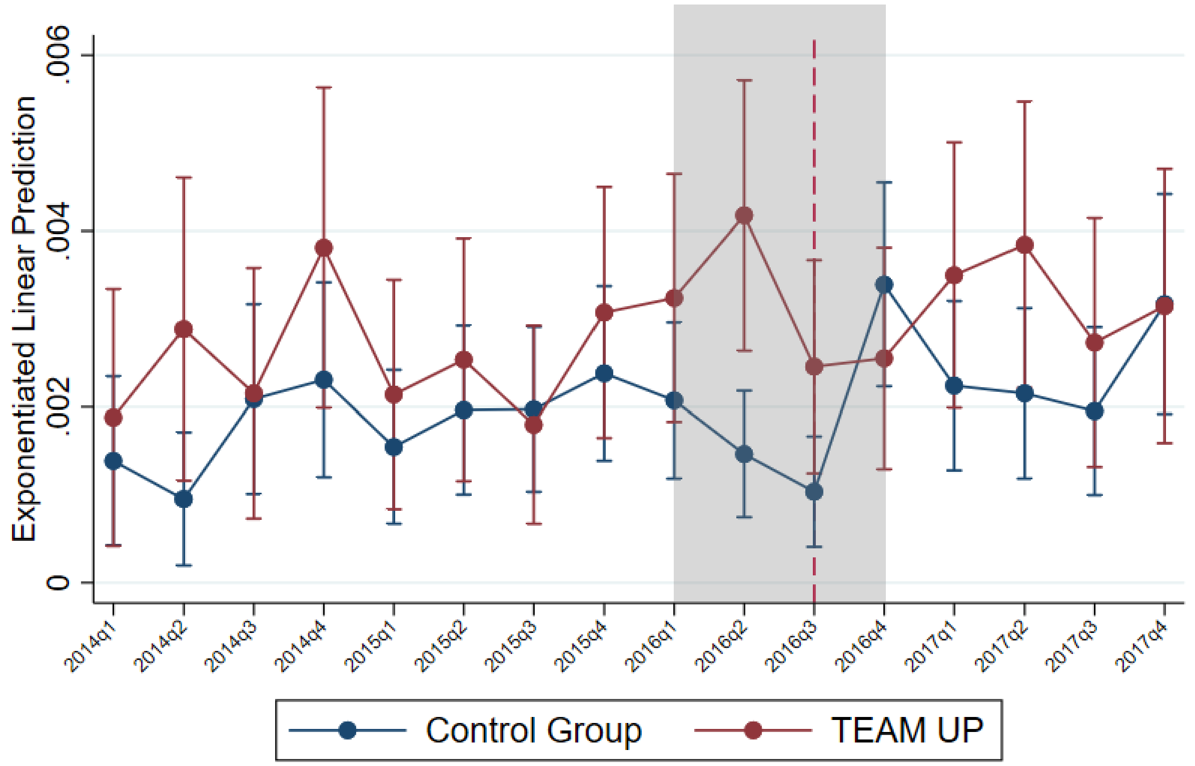

Note: This graph shows time trend in inpatient admissions with MH DX among Medicaid-enrolled children.

© 2023 Kim J et al. *JAMA Network Open.*

eFigure 3. Time Trends in Emergency Department Visits Among Medicaid-Enrolled Children

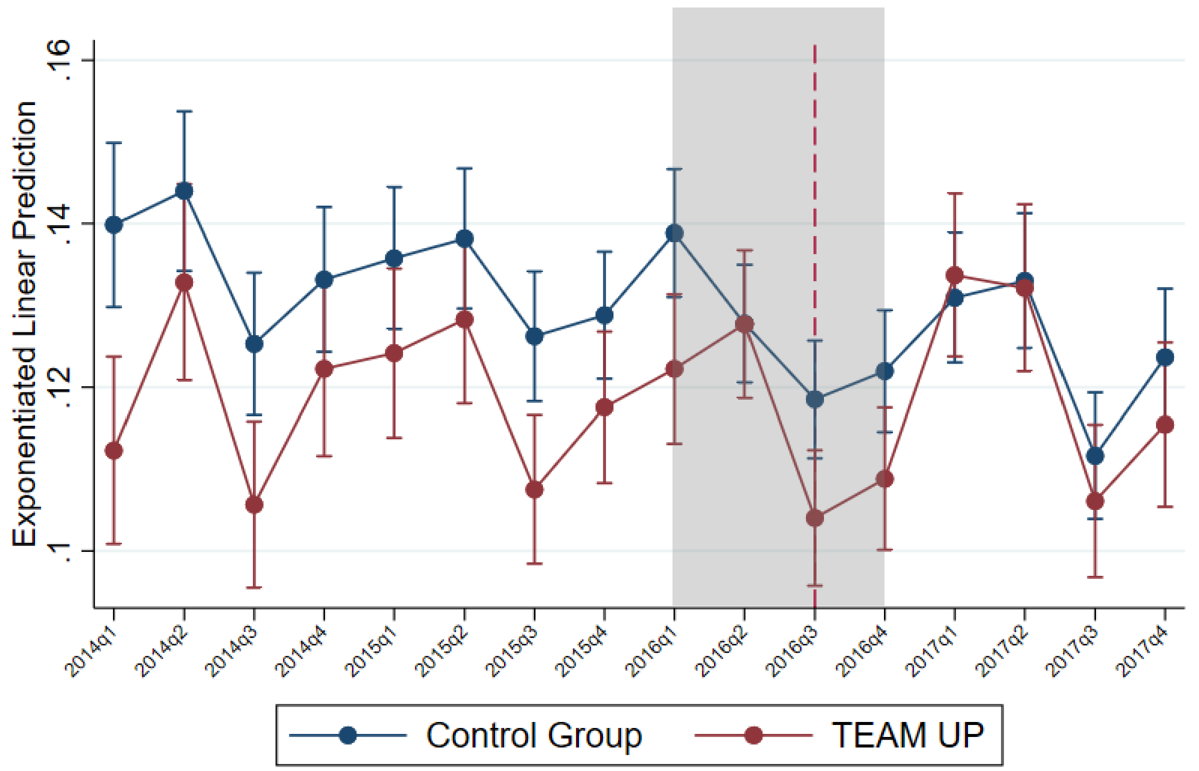

Note: This graph shows time trend in emergency department visits among Medicaid-enrolled children.

© 2023 Kim J et al. *JAMA Network Open.*

eFigure 4. Time Trends in Emergency Department Visits Among Medicaid-Enrolled Children With a Baseline Mental Health Diagnosis

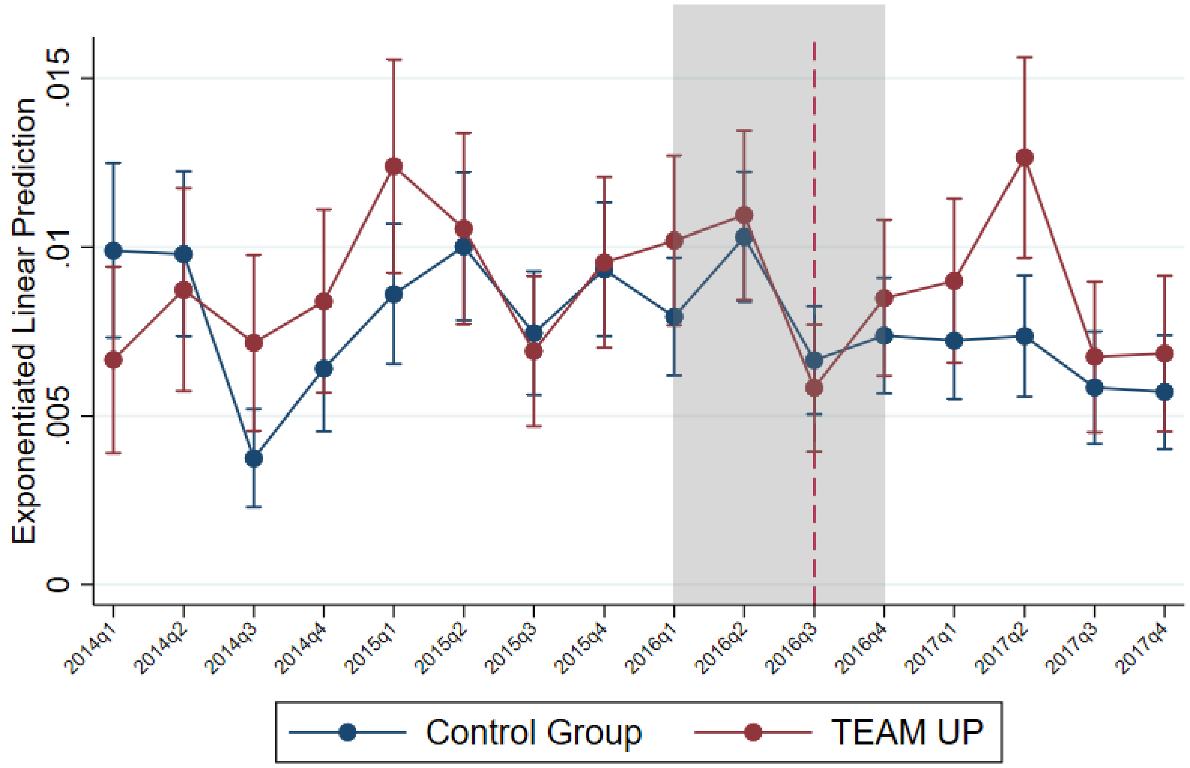

Note: This graph shows time trend in emergency department visits with MH DX among Medicaid-enrolled children.

© 2023 Kim J et al. *JAMA Network Open.*

eFigure 5. Time Trends in Primary Care Visits Among Medicaid-Enrolled Children

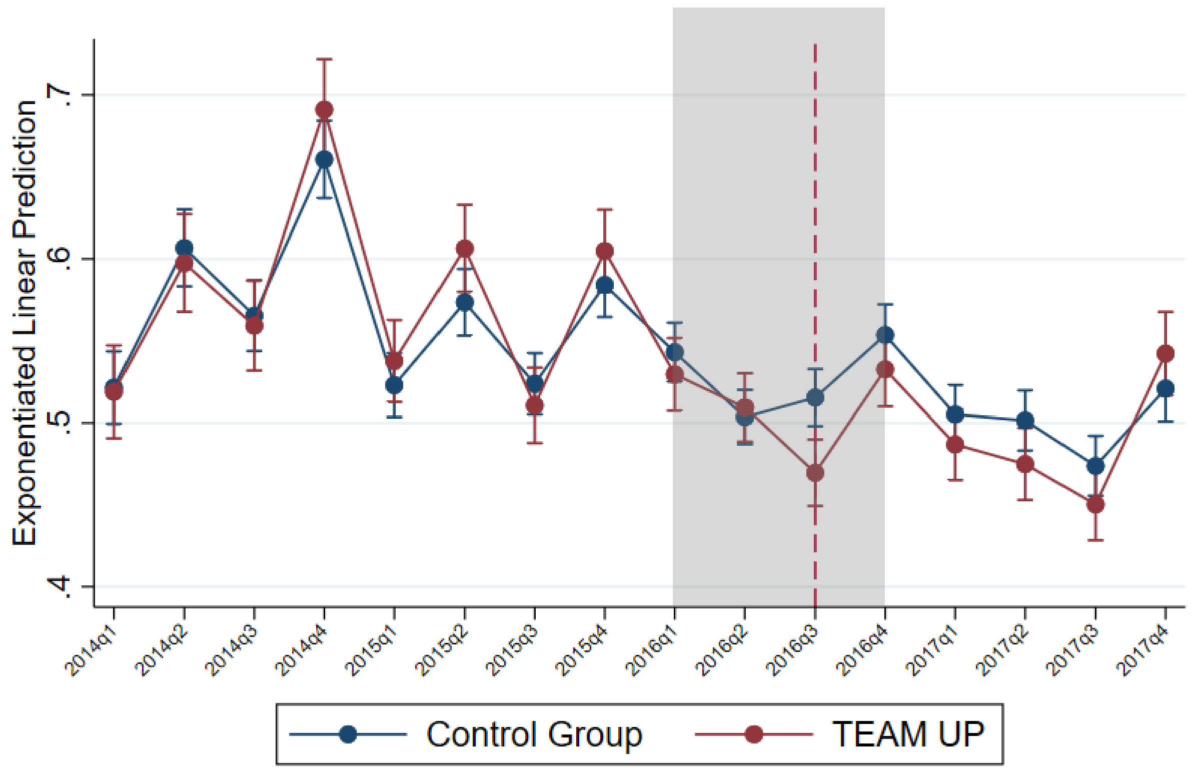

Note: This graph shows time trend in primary care visits among Medicaid-enrolled children.

© 2023 Kim J et al. *JAMA Network Open.*

eFigure 6. Time Trends in Primary Care Visits Among Medicaid-Enrolled Children With a Baseline Mental Health Diagnosis

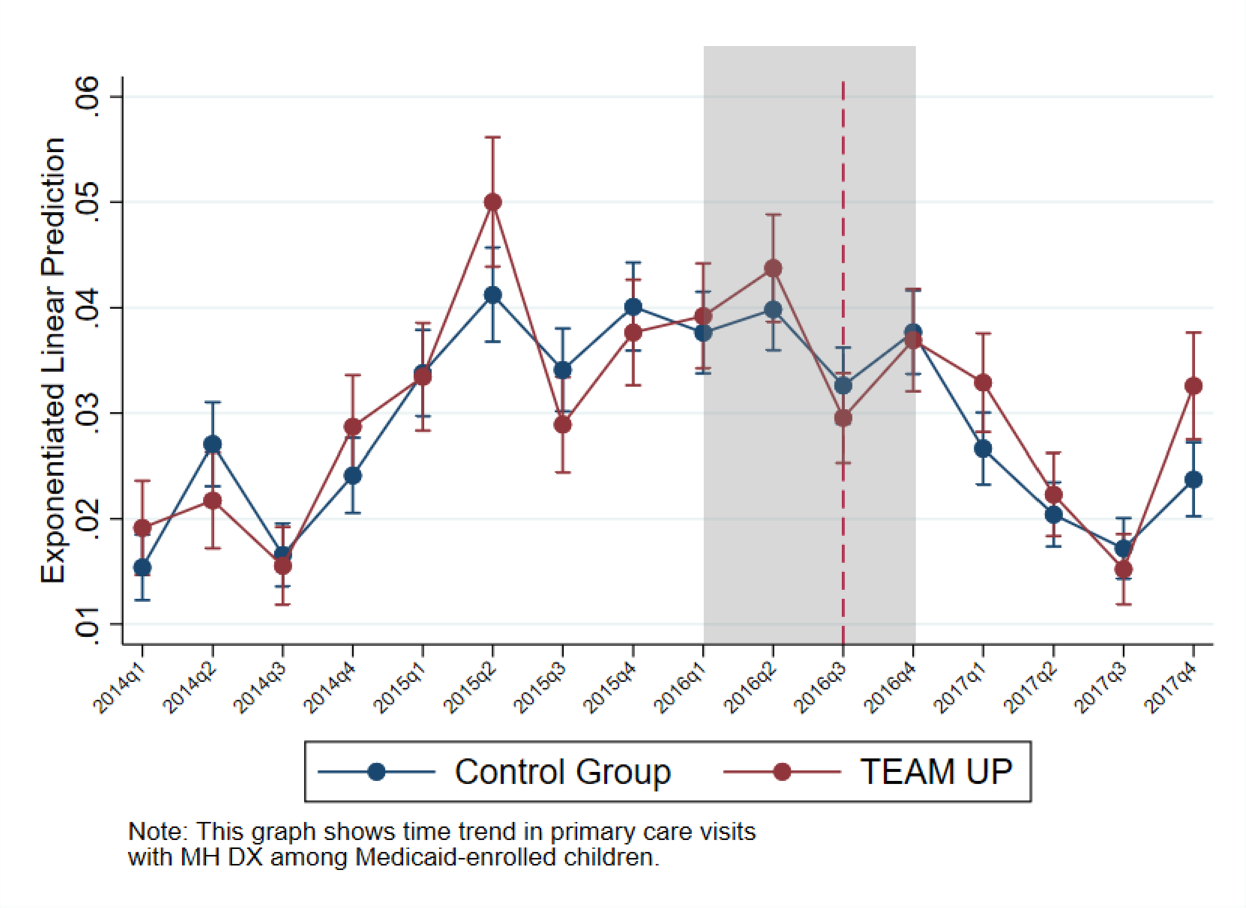

© 2023 Kim J et al. *JAMA Network Open.*

eFigure 7. Time Trends in Any Mental Health Service Utilization Among Medicaid-Enrolled Children

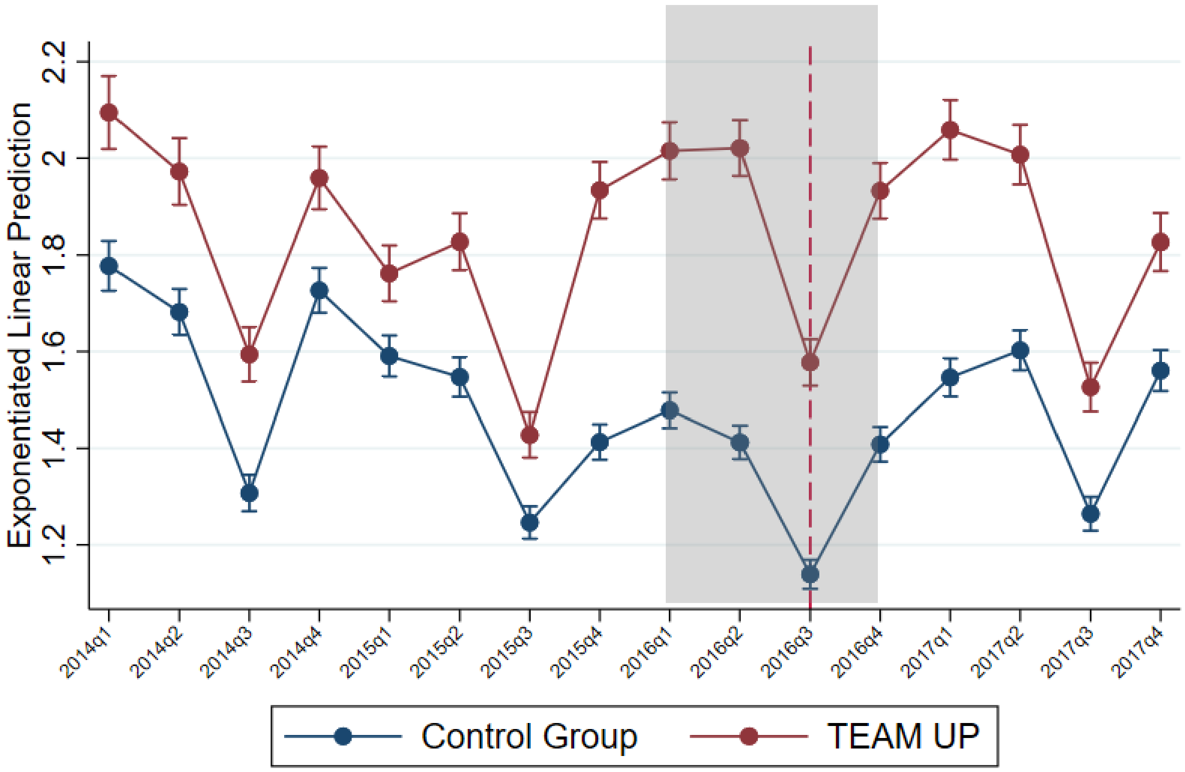

Note: This graph shows time trend in any mental health service utilization among Medicaid-enrolled children.

© 2023 Kim J et al. *JAMA Network Open.*

eFigure 8. Time Trends in Mental Health Therapy Utilization Among Medicaid-Enrolled Children

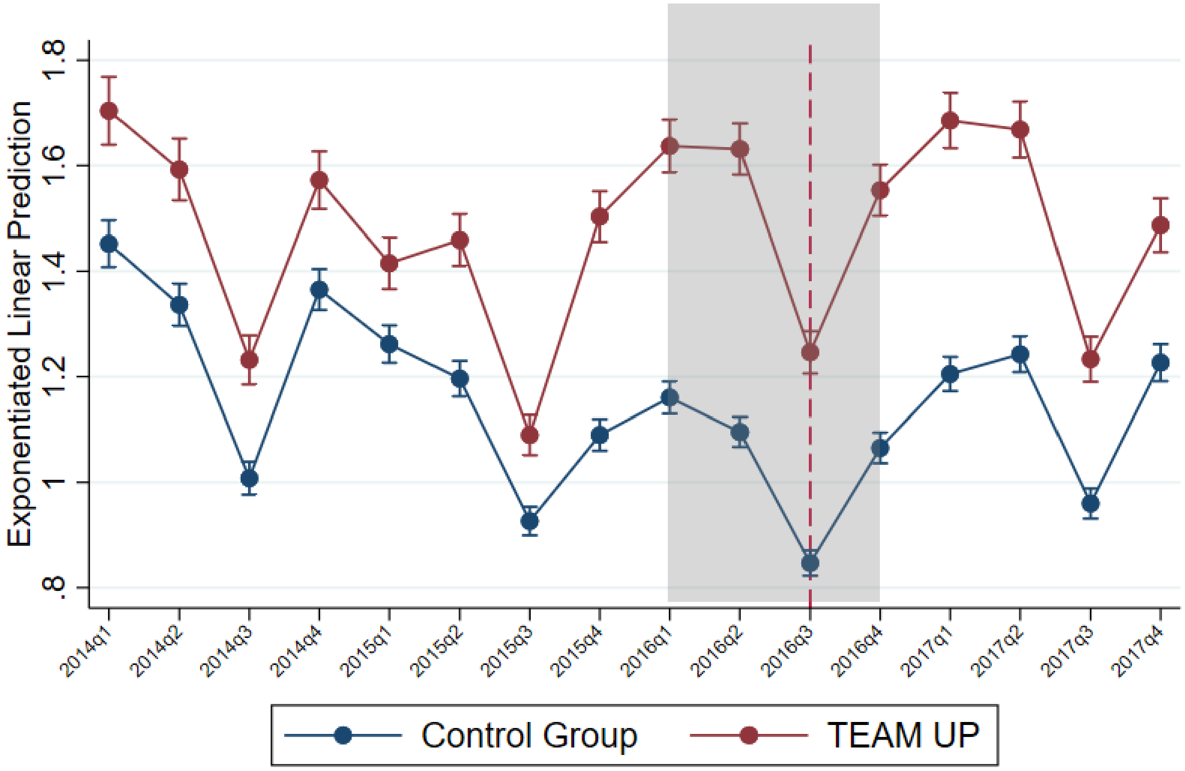

Note: This graph shows time trend in mental health therapy utilization among Medicaid-enrolled children.

© 2023 Kim J et al. *JAMA Network Open.*

eFigure 9. Time Trends in Mental Health Consultation Utilization Among Medicaid-Enrolled Children

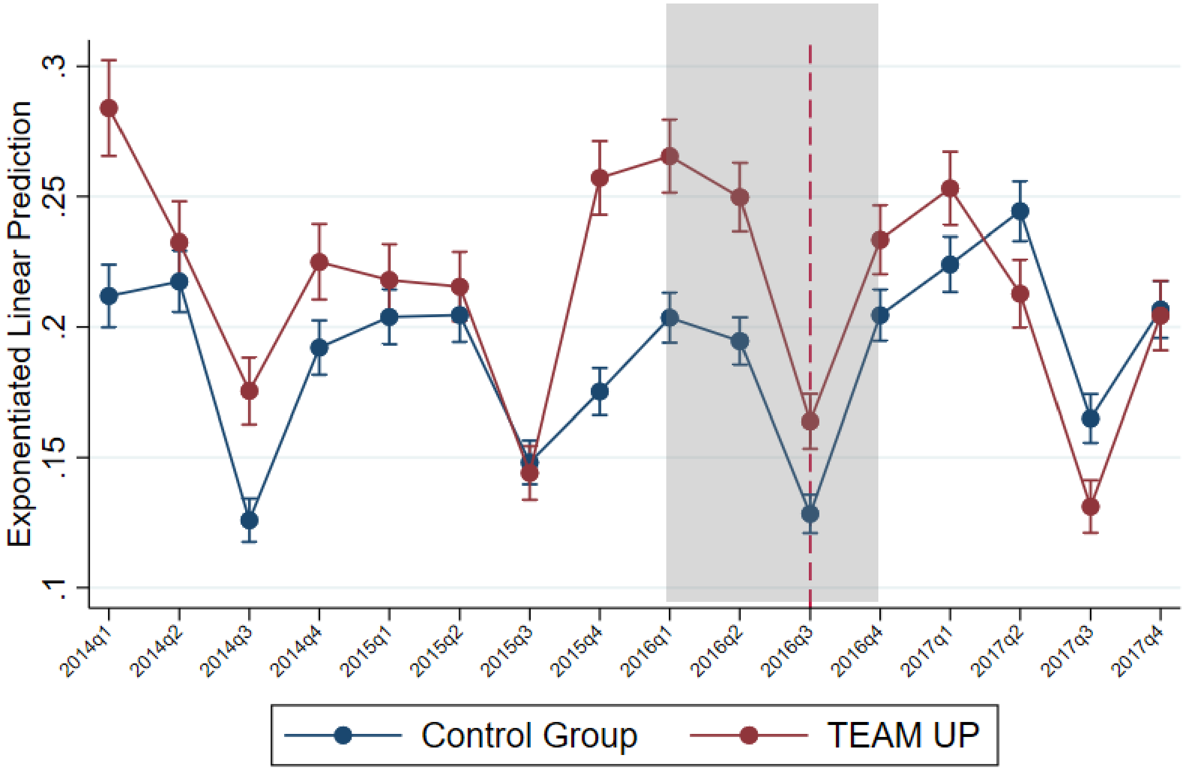

Note: This graph shows time trend in mental health consultation utilization among Medicaid-enrolled children.

© 2023 Kim J et al. *JAMA Network Open.*

eFigure 10. Time Trends in Mental Health Testing Utilization Among Medicaid-Enrolled Children

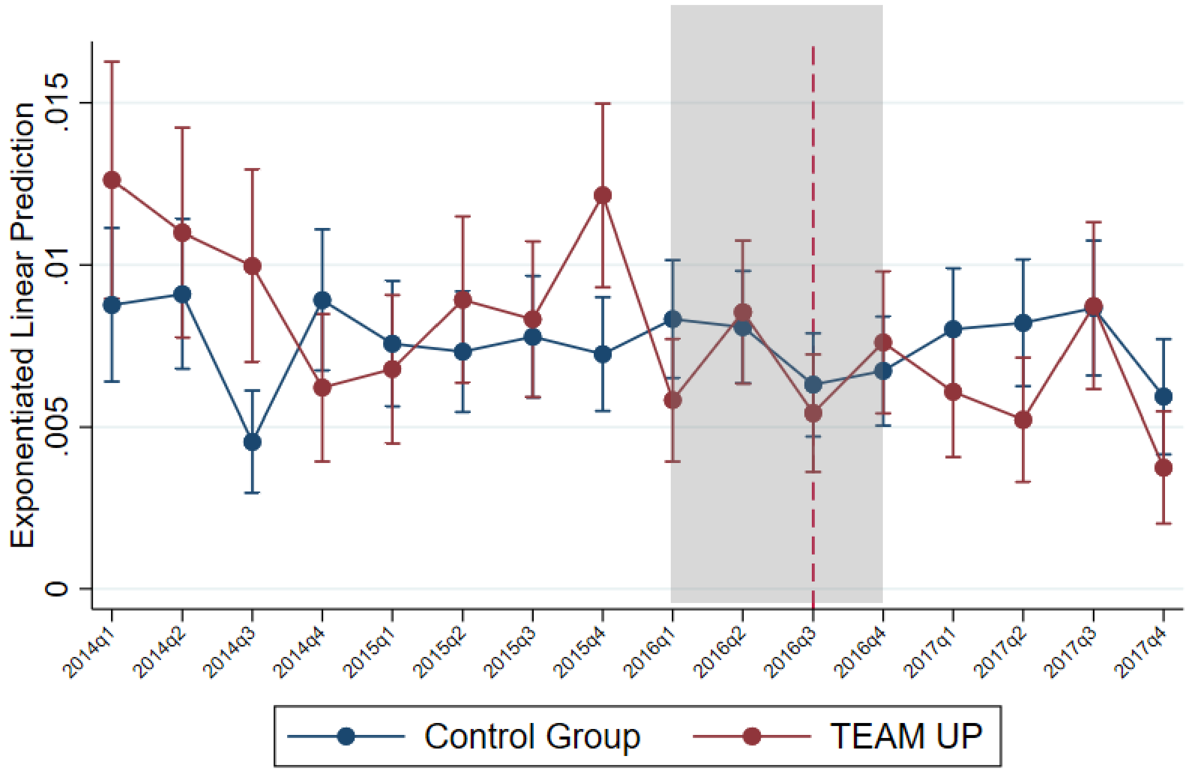

Note: This graph shows time trend in mental health testing utilization among Medicaid-enrolled children.

© 2023 Kim J et al. *JAMA Network Open.*

eFigure 11. Time Trends in Mental Health Screening Utilization Among Medicaid-Enrolled Children

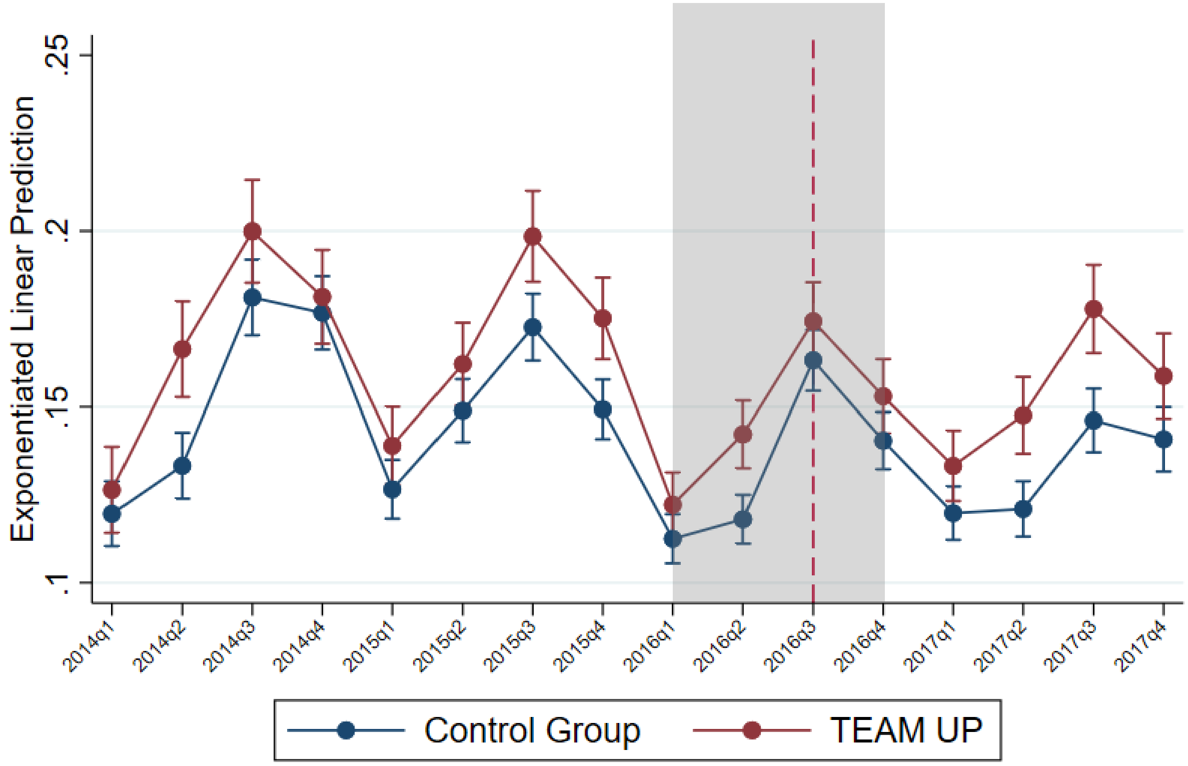

Note: This graph shows time trend in mental health screening utilization among Medicaid-enrolled children.

© 2023 Kim J et al. *JAMA Network Open.*

eFigure 12. Time Trends of Any Psychotropic Medication Use (>0% of Days in Quarter) Among Medicaid-Enrolled Children

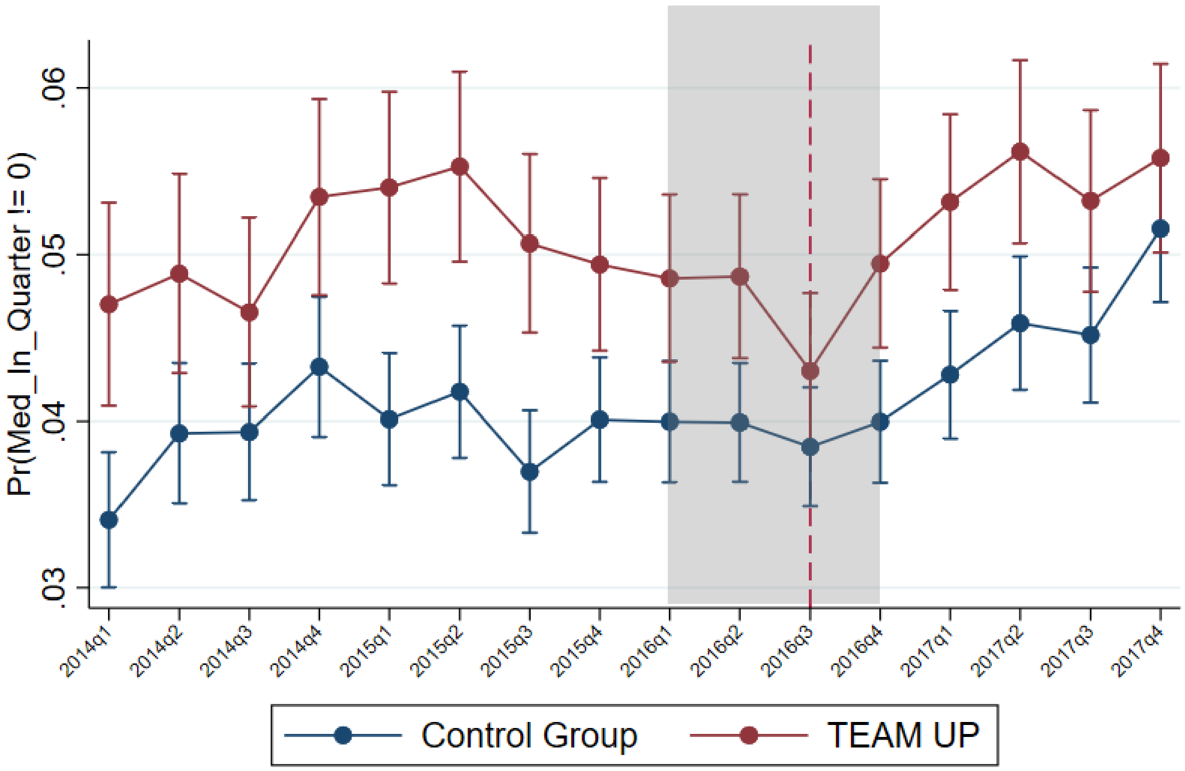

Note: This graph shows time trend in any psychotropic medication use (>0% of days in quarter) among Medicaid-enrolled children.

© 2023 Kim J et al. *JAMA Network Open.*

eFigure 13. Time Trends of Any Stimulant Use Among Medicaid-Enrolled Children

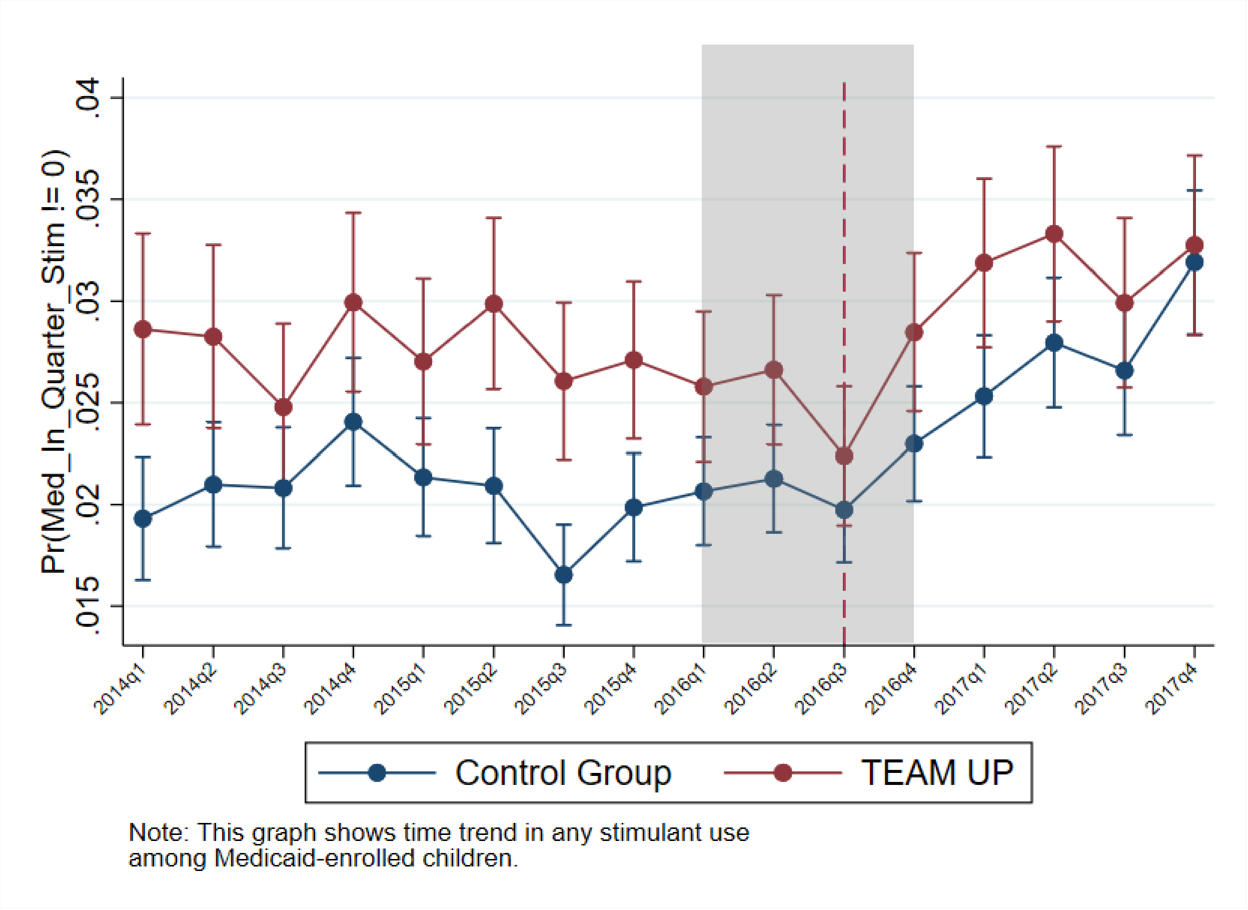

© 2023 Kim J et al. *JAMA Network Open.*

eFigure 14. Time Trends of Polypharmacy (Having > 1 Class of Psychotropic Medication) Among Medicaid-Enrolled Children

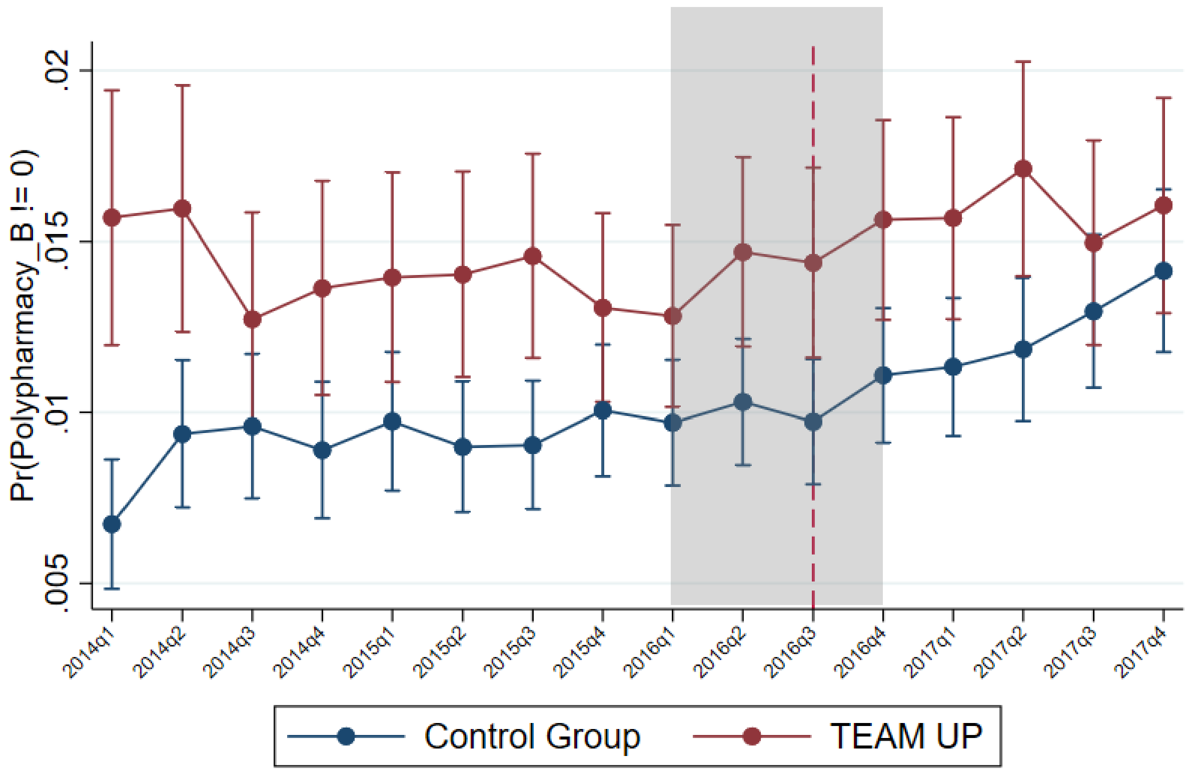

Note: This graph shows time trend in polypharmacy (having > 1 class of psychotropic medication) among Medicaid-enrolled children.

© 2023 Kim J et al. *JAMA Network Open.*

eFigure 15. Time Trends of 7-Days FU After ED Visit for MH/Intentional Self-Harm DX (Primary)

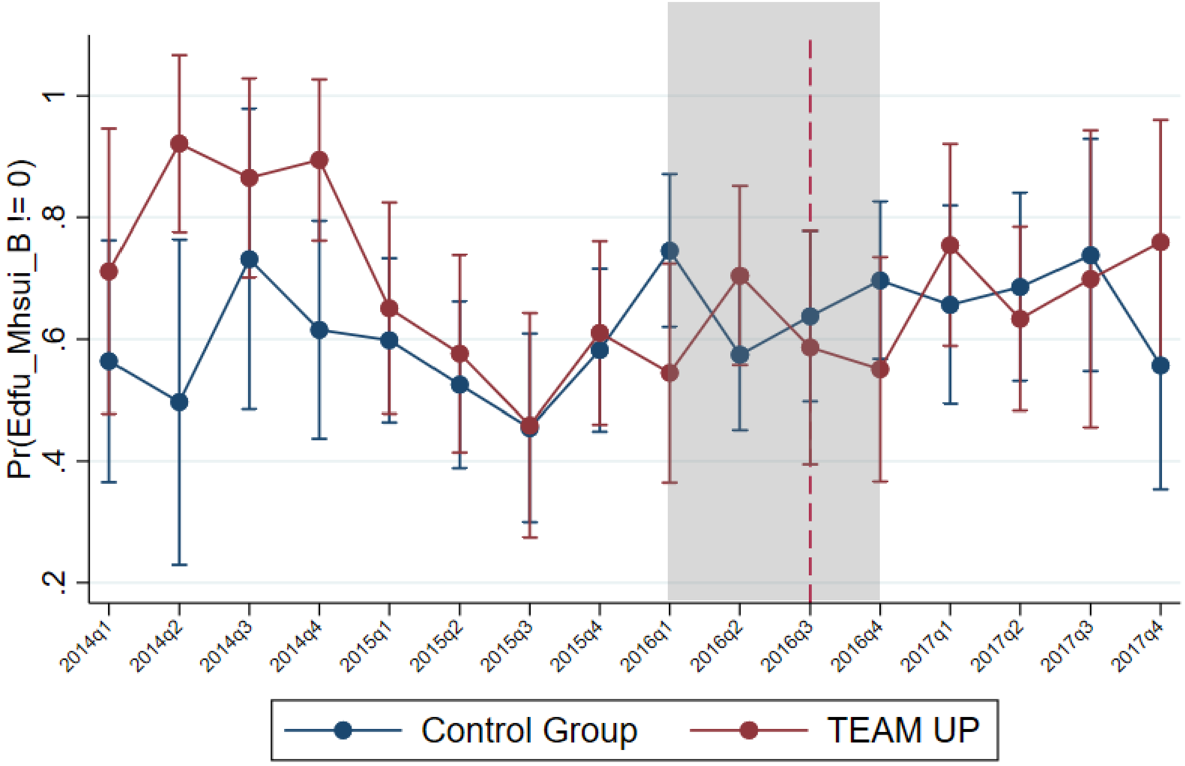

Note: This graph shows time trend in 7-days follow-up after emergency department visit for mental health/intentional self-harm primary diagnoses among Medicaid-enrolled children.

© 2023 Kim J et al. *JAMA Network Open.*

eFigure 16. Time Trends of 7-Days FU After Hospitalization for MH DX (Primary)

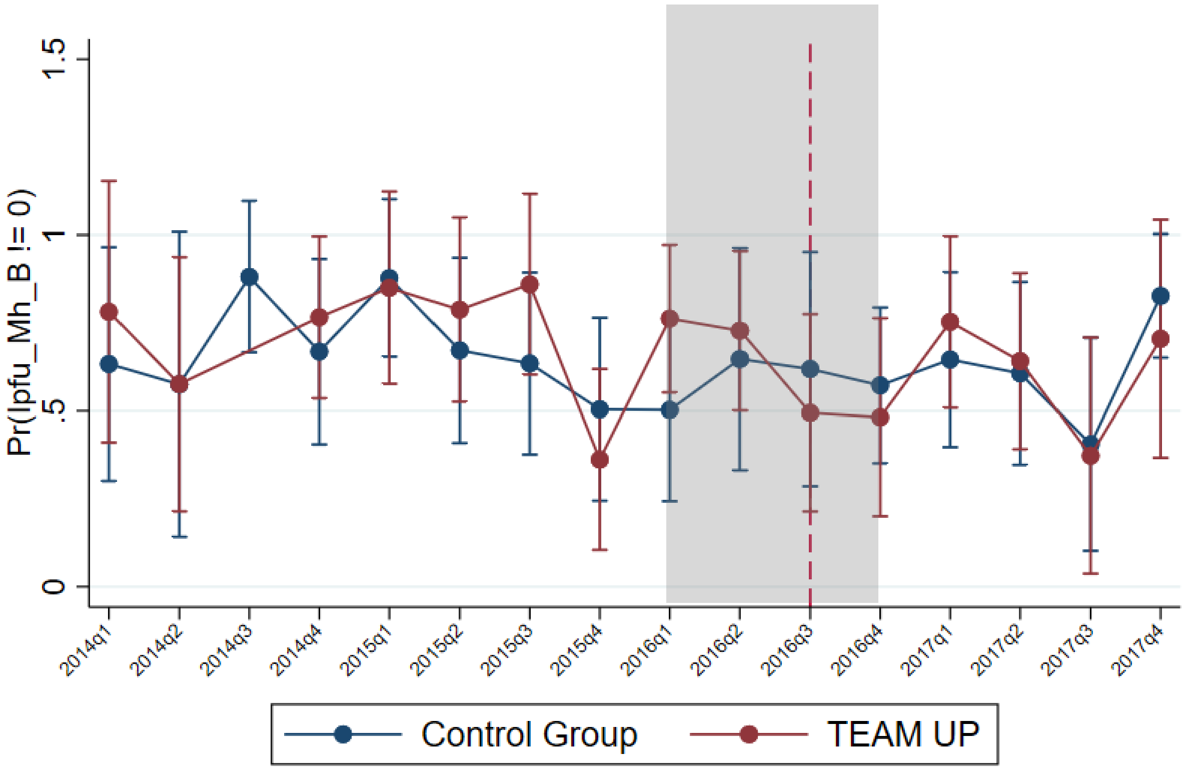

Note: This graph shows time trend in 7-days follow-up after hospitalization for mental health primary diagnoses among Medicaid-enrolled children.

© 2023 Kim J et al. *JAMA Network Open.*
