# Supplementary material for: Association of Integrating Mental Health Into Pediatric Primary Care at Federally Qualified Health Centers With Utilization and Follow-up Care
Source: JAMA Netw Open. 2023 Apr 26;6(4):e239990. doi: 10.1001/jamanetworkopen.2023.9990 (PMC10134009; doi:10.1001/jamanetworkopen.2023.9990)
Supplement: Supplement 2. — Data Sharing Statement [file jamanetwopen-e239990-s002.pdf]

Data Sharing Statement

Kim. Association of Integrating Mental Health Into Pediatric Primary Care at Federally Qualified Health Centers With Utilization and Follow-up Care. *JAMA Netw Open*. Published April 26, 2023. doi:10.1001/jamanetworkopen.2023.9990

Data
Data available: No

Additional Information
Explanation for why data not available: Per our DUA with the state, we cannot share these claims data. All interested researchers must obtain data through CHIA and have appropriate data management plan and security requirements in place.
